# Plasma-lyte solution versus saline in kidney transplantation: A systematic review and meta-analysis of randomized controlled trials

**DOI:** 10.1371/journal.pone.0320082

**Published:** 2025-04-09

**Authors:** Yucai Chang, Yuechen Qin, Yue Zou, Haijian Zeng, Chunlan Li, Mengtian Qin, Jianyu Wu, Jian Ban

**Affiliations:** The First Affiliated Hospital of Guangxi University of Science and Technology, Guangxi University of Science and Technology, Liuzhou, Guangxi Province, China; Universidad Mayor de San Simon Facultad de Medicina, BOLIVIA, PLURINATIONAL STATE OF

## Abstract

**Introduction:**

The optimal intravenous fluid for kidney transplantation (KT) is still controversial. This meta-analysis aimed to compare the efficacy and safety of plasma-lyte solution (PL) versus saline (NS) in kidney transplantation.

**Materials and methods:**

A comprehensive search was conducted across four databases (PubMed, Embase, Web of Science, and the Cochrane Library) to identify relevant randomized controlled trials (RCTs) comparing plasma-lyte and saline in kidney transplantation. Risk of bias was assessed using the Cochrane RoB 2.0 tool. Meta-analyses of delayed graft function (DGF), creatinine levels, urine output, blood pH, bicarbonate, base excess, electrolytes, serum chloride, potassium and sodium immediately post-surgery were performed.

**Results:**

Totally six randomized controlled studies with 1197 patients were included. In comparison to the NS group, the PL group exhibited a significantly lower incidence of DGF (OR: 0.66, 95% CI: 0.51 to 0.86, P =  0.002) and reduced serum chloride (MD: -8.87, 95% CI: -13.50 to -4.25, P = 0.0002) as well as serum sodium(MD: -1.08, 95% CI: -1.54 to -0.61, P <  0.00001), while blood pH(MD: 0.05, 95% CI: 0.03 to 0.07, P <  0.00001), bicarbonate (MD: 2.09, 95% CI: 1.21 to 2.97, P = 0.005), and base excess levels (MD: 2.42, 95% CI: 0.72 to 4.11, P <  0.00001) were significantly elevated. No statistically significant differences were observed in creatinine, urine output, or potassium concentrations between two groups.

**Conclusions:**

This meta-analysis compared the efficacy and safety between plasma-lyte and saline in kidney transplantation. Plasma-lyte reduced delayed graft function in kidney transplant compared to saline.

**Trial registration:**

PROSPERO (CRD42024588701).

## 1. Introduction

KT is considered the preferred treatment for patients with end stage kidney disease (ESKD), significantly improving their quality of life and providing a median survival rate comparable to that of individuals without ESKD [1]. While KT provides eligible patients with ESKD the best opportunity for long-term, dialysis-free survival at the lowest cost to the health care system, maximizing transplant access, reducing access disparities, and optimizing long-term allograft survival are ongoing challenges [2]. Among adult recipients of deceased-donor kidney transplants (DDKT) from 2015 to 2017, 5-year graft survival was lowest among older recipients, with a survival rate of 67.8% at 5 years for those aged 65 or older, compared to 81.4% for recipients aged 18-34 years. In contrast, among adult recipients of living-donor kidney transplants (LDKT) during the same period, the 5-year graft survival rate was 80.8% for those aged 65 or older, compared to 90.0% for recipients aged 18-34 years [3].

Patients undergoing KT are subject to a wide variety of intraoperative complications including hemodynamic instability, acid-base and electrolyte disturbances because of impaired renal function, and co-morbid diseases [4]. Maintenance of intravascular volume during KT is crucial to ensure optimal graft perfusion and function [5]. Intraoperative fluid management may affect the outcome after KT [6].

Substantial controversy remains about the optimal choice and combination of fluid to be given during the perioperative period. A recent consensus statement by the American Society of Anesthesiologists committee established that the group treated with normal 0.9% saline (NS) had elevated potassium levels, more frequent treatment of hyperkalemia, and more severe acidosis in comparison to the group treated with plasma-lyte (PL) [6]. Historically, NS has been the conventional intravenous fluid used in KT. Nevertheless, the safety of the treatment has been questioned because of several documented serious side effects, including severe hyperchloremia, metabolic acidosis, hyperkaliemia, and the need for dialysis following dialysis [6–8]. Several randomized controlled trials (RCTs) have assessed PL as a prospective substitute [7–10]. A significant discovery from the recent best-Fluids trial, which included 808 patients who underwent KT from deceased donors, indicated that the incidence of delayed graft function (DGF) was 30% in the group receiving a PL and 40% in the group receiving an NS (P <  0.0001), recommending PL as the preferred intravenous infusion after KT [8]. DGF represents a type of acute kidney injury that is relatively reversible and can occur postoperatively, with or without oliguria/anuria, necessitating at least one dialysis session within the initial seven days post-transplantation [11]. The early recovery status of DGF significantly contributes to enhancing post-transplant management [12]. Therefore, we conducted a meta-analysis to compare the efficacy and safety between PL and NS in KT.

## 2. Materials and methods

### 2.1. Search strategy

The current meta-analysis adhered to the 2020 guidelines set by the Preferred Reporting Project for Systematic Review and Meta-Analysis (PRISMA). The present meta-analysis has been officially recorded at PROSPERO under the registration number CRD42024588701. A systematic search was conducted in four databases, namely PubMed, Embase, Web of Science, and the Cochrane Library, for literature published until August 2, 2024. The search strategy followed the PICOS principle and involved a combination of Mesh terms and free-text words. The specific search strategy used was: “kidney transplantation” AND “Plasma-Lyte” AND “Normal Saline” AND “RCT”. Supplementary material 1 provided a comprehensive listing of the search results.

### 2.2. Inclusion and exclusion criteria

The criteria for inclusion were as follows: (1) Participant: patients with ESKD due to any cause, undergoing KT, whether receiving a transplant from living or deceased donor; (2) Intervention: perioperative PL regardless of infusion rate and volume; (3) Control: perioperative NS regardless of rate and volume of infusion; (4) Outcomes: at least one of the following outcomes is documented: the occurrence of DGF, evaluations of renal function (creatinine levels and urine output) on postoperative days (POD) 1, 2, and 7, blood pH, bicarbonate, base excess, electrolytes serum chloride, potassium, and sodium immediately post-surgery. (5) Study design: RCT.

The criteria for exclusion were as follows: (1) different types of articles, such as case reports, publications, letters, comments, reviews, meta-analyses, editorials, animal studies, protocols, conference, etc.; (2) other types of malignancies or diseases; (3) not relevant; (4) full text not available; (5) duplicate patient cohort. (6) non-RCT.

### 2.3. Literature selection

The literature selection process entailed the elimination of duplicate entries and was facilitated by the use of EndNote (Version X9; Clarivate Analytics). The initial search was conducted by two independent reviewers who meticulously eliminated duplicate records, evaluated the relevance of titles and abstracts, and systematically classified each study as either included or excluded. Discrepancies were resolved through consensus. In instances where agreement was not achieved, a third reviewer intervened as a mediator. The screening process was executed in two distinct phases: (i) an initial screening of titles and abstracts to ascertain the relevance of studies to this meta-analysis; (ii) a subsequent full-text screening to evaluate final eligibility according to predefined inclusion criteria for both qualitative and quantitative analyses.

### 2.4. Data extraction

The evaluator conducted independent data collection and extraction into a standardized data extraction Excel spreadsheet. The extracted data encompassed the characteristics of the included studies: name of the first author, year of publication, country, study design, total number of participants, type of intervention, control conditions, inclusion criteria, primary outcomes, and combined interventions. Baseline characteristics of the study subjects included the number of subjects, average age, sex, etiology of ESKD, and type of preoperative dialysis method; Intraoperative variables included cold ischemia time, total fluid infusion volume, and operation duration; The efficacy and safety indicators comprised DGF, renal function (creatinine levels and urine output) on POD 1, 2, and 7, as well as blood pH, bicarbonate, base excess, electrolytes, and serum chloride, potassium, and sodium immediately post-surgery. One group of randomized controlled trials included in this review did not report DGF, and we were unable to contact the authors by email to obtain DGF. For the absence of data on Secondary outcomes, we also contacted the authors by email, but did not receive a response. Any discrepancies were resolved by consensus among the reviewers.

### 2.5. Risk of bias

Two autonomous reviewers evaluated the quality assessment of the trials that were included. The risk of bias for selected RVTs was assessed using the Cochrane RoB 2.0 tool [13]. The domains that were evaluated included the risk of bias resulting from the randomization process, the risk of bias due to deviation from the intended intervention, the risk of bias due to missing outcome data, the risk of bias in the measuring of outcomes, and the risk of bias in selecting the reported results.

### 2.6. Statistical analysis

Statistical analysis was performed utilizing Review Manager v5.4 software. We employed odds ratios (OR) for binary outcomes and mean differences (MD) for continuous outcomes, both shown with 95% confidence intervals (CI), within the basic structure of the fixed-effects model. The medians and interquartile ranges were converted into means and standard deviations for the continuous data. In instances of considerable heterogeneity, as identified by the χ^2^ test (P <  0.1) or marked heterogeneity signified by the I^2^ test (values ranging from 50 to 100%), we utilized the random-effects model to accommodate variability. A preplanned subgroup analysis was conducted based on the type of balanced crystalloid and the type of donor. A leave-one-out sensitivity analysis was done due to considerable heterogeneity. The publishing bias was assessed through a visual examination of the funnel plot [14].

## 3. Results and analysis

### 3.1. Search results

**Fig 1:**
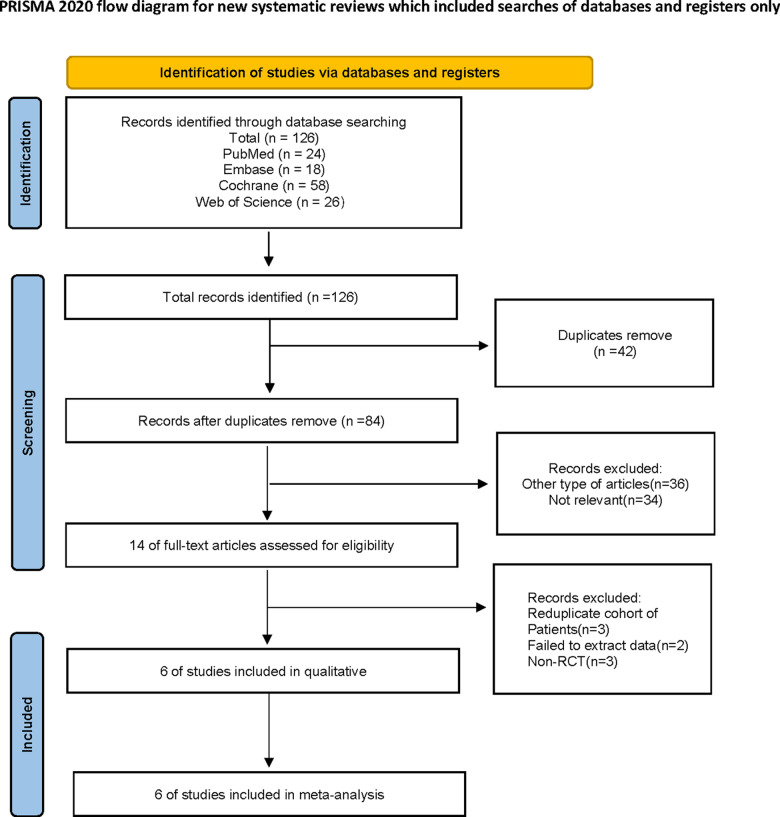
Flow chart of literature search strategies.

Fig 1 illustrates the process of selecting and integrating literature. The search process yielded a total of 126 articles initially. After excluding duplicate entries and reviewing the titles as well as abstracts of the remaining articles for relevance, 26 studies were selected for full-text review. Finally, six RCTs met the inclusion criteria for our systematic review and meta-analysis.

### 3.2. Characteristics of included studies and participants

Totally six randomized controlled studies with 1197 patients were included [7–10,15,16]. Patients in three RCTs received DDKT [7,8,15], while patients in another three RCTs received LDKT [9,10,16]. Based on the specific treatments, patients were categorized into two subgroups: Group A receiving PL and Group B receiving NS. Detailed summaries of the characteristics of the included RCTs and the baseline characteristics of the participants are presented in Table 1.

**Table 1:** Patient characteristics of included studies and patients.

Author, year	Study type	Country	Type of donor	Group	cases	Mean age (years)	Male (%)	Mean BMI (kg/m^2^)	Cold ischemic time	Operation time, m	Fluid volume, mL
Collins et al. 2023	Pragmatic, registry embedded, multicenter, double-blind, RCT	Australia and New Zealand	Deceased	PL NS	404 403	55 54	260 (64%) 252 (62%)	27 27	NA NA	NA	8143 (4077) 7180 (3448)
do Nascimento Junior et al. 2022	Single-blinded, RCT	Brazil	Deceased	PL NS	50 51	45.9 47.2	30 (60%) 32 (62.7%)	25.43 27.16	23 h 23 h	NA	1,628 (595) 1,627 (532)
L. Weinberg 2017	RCT	Australia	Deceased	PL NS	24 25	53 49	15 (63%) 18 (72%)	29.5 27.0	10.8 (9.8, 13.2) h 11.8 (6.3; 14.3) h	212 (39)	2500 (2000, 3000) 3000 (2000,3000)
Hadimioglu et al. 2008	Prospective, double blinded, RCT	Turkey	Living	PL NS	30 30	37.6 37.7	NR NR	22.67 23.44	33.9(7.3) m 31.5(8.1) m	208 (42)	2756 (800) 2868 (780)
Kim et al. 2013	Double-blinded, RCT	Korea	Living	PL NS	30 30	44 46	17 (56.7%) 21 (70%)	22.68 22.63	NA NA	180 (156–198)	3083 (1082) 3249 (891)
Saini et al. 2021	Prospective, double blinded, RCT	India	Living	PL NS	60 60	46.35 44.13	NR NR	23.13 21.67	28.20 ± 4.36 m 30.73 ± 31.30 m	168 (150–192)“	1335.83 ± 189.13 1895 ± 290.19

BMI, Body Mass Index; NS, normal saline; PL, plasma-lyte; RCT, randomized controlled trial; NA, not available.

### 3.3. Risk of bias

**Fig 2:**
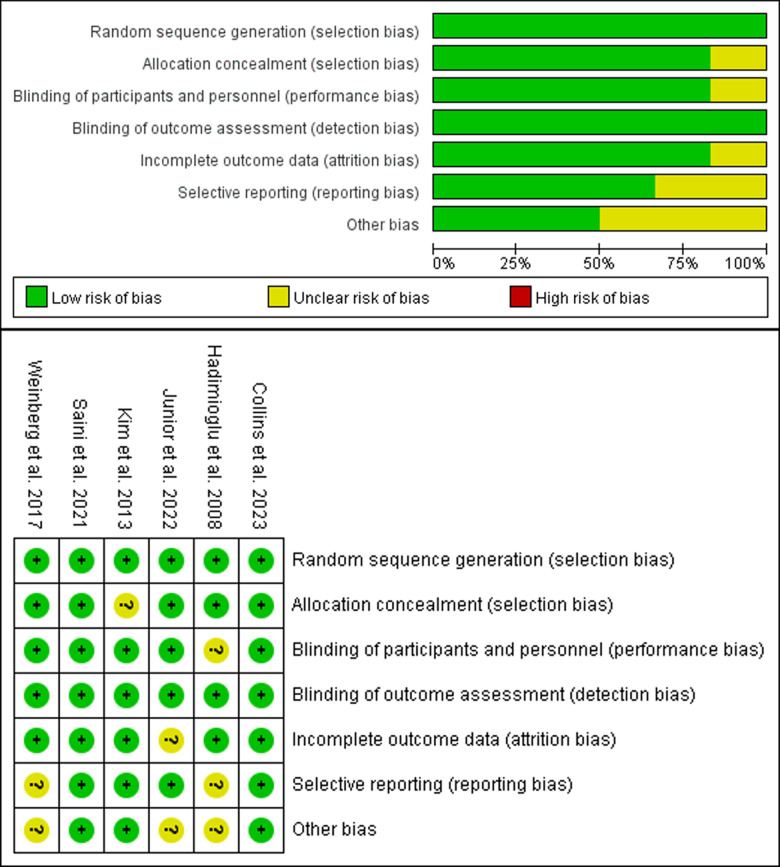
Risk of bias assessment diagram.

Fig 2 provides a succinct summary of the risk of bias assessment results by the Cochrane RoB 2.0 tool. Six trials provided adequate randomization sequences, six reported appropriate allocation concealment, five explicitly conducted blinding of participants, five reported blinding of outcome assessors, six did not perform selective reporting, and six did not show any other bias.

### 3.4. Primary outcome (DGF)

**Fig 3:**
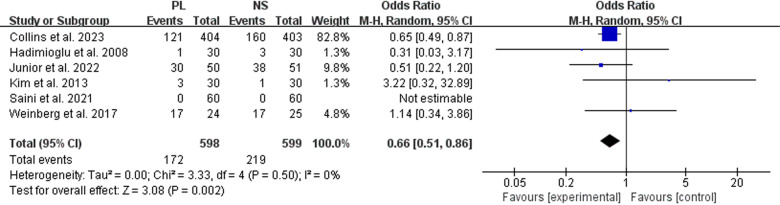
Forest plot of the meta-analysis for DGF.

Six studies documented the DGF. Our meta-analysis demonstrated that the risk of DGF was significantly reduced in the PL group compared to the NS group (OR: 0.66, 95% CI: 0.51 to 0.86, P =  0.002) (Table 2 and Fig 3). The results indicated homogeneity (P =  0.50, I² =  0%).

**Table 2:** Results of the meta-analysis.

Outcomes	No. of studies	Sample size		Overall effect size	95% CI of overall effect	P value	Heterogeneity	
		PL	NS				I^2^ (%)	P value
Delayed graft function	6	598	599	OR = 0.66	0.51, 0.86	0.002	0	0.50
POD1 serum creatinine	5	548	548	MD = -0.08	-0.17, -0.34	0.53	0	0.86
POD2 serum creatinine	5	547	547	MD = − 0.01	-0.22, 0.25	0.90	0	0.71
POD7 serum creatinine	5	542	533	MD = -0.07	-0.28, 0.14	0.53	3	0.39
POD1 urine output	4	144	145	MD = -1.22	-3.39, 0.96	0.27	90	<0.00001
POD2 urine output	5	548	548	MD = -0.11	-1.03, 0.80	0.81	79	0.00009
POD7 urine output	3	120	120	MD = -0.17	-0.54, 0.19	0.35	0	0.80
Blood pH	6	500	490	MD = 0.05	0.03, 0.07	<0.00001	72	0.003
Bicarbonate levels	6	579	587	MD = 2.09	1.21, 2.97	<0.00001	78	0.0003
Base excess	4	162	163	MD = 2.42	0.72, 4.11	0.005	88	<0.001
Serum chloride	5	578	583	MD = -8.87	-13.50, -4.25	0.0002	98	<0.00001
Serum sodium	4	497	499	MD = -1.01	-1.46, -0.57	<0.00001	0	0.43
Serum potassium	5	557	558	MD = -0.03	-0.20, 0.14	0.73	64	0.03

PL, plasma-lyte solution; NS, normal saline; OR, odd ratio; MD, mean difference; POD, postoperative days.

In subgroup analysis of LDKT, there was no significant difference between two group regarding DGF (OR: 1.00, 95% CI: 0.10 to 9.91, P =  1.00). While in subgroup analysis of DDKT, the risk of DGF was significantly reduced in the PL group compared to the NS group (OR: 0.65, 95% CI: 0.50 to 0.85, P =  0.002) (Table 3).

**Table 3:** Results of the subgroup analysis.

Outcomes	Type of donor	No. of studies	Sample size		Overall effect size	95% CI of overall effect	P Value	Heterogeneity		Test for subgroup differences
			PL	NS				I^2^(%)	P value	P value
Delayed graft function	Living	3	120	120	OR = 1.00	0.10,9.91	1.00	49	0.16	0.72
	Deceased	3	478	479	OR = 0.65	0.50,0.85	0.002	0	0.57	
POD1 serum creatinine	Living	3	120	120	MD = 0.00	-0.48, 0.48	0.99	0	0.58	0.70
	Deceased	2	428	428	MD = 0.11	-0.19, 0.41	0.46	0	0.8	
POD2 serum creatinine	Living	3	120	120	MD = 0.09	-0.20, 0.39	0.54	0	0.92	0.48
	Deceased	2	427	427	MD = -0.26	-1.20, 0.67	0.58	24	0.25	
POD7 serum creatinine	Living	3	120	120	MD = 0.04	-0.20, 0.27	0.74	0	0.82	0.08
	Deceased	2	422	413	MD = -0.39	-0.80, 0.02	0.06	0	0.82	
POD1 urine output	Living	3	120	120	MD = -2.14	-3.78, -0.50	0.01	76	0.02	0.001
	Deceased	1	25	25	MD = 1.22	-3.39, 0.96	0.05		–	
POD2 urine output	Living	3	120	120	MD = -0.87	-1.74, 0.08	0.07	43	0.17	0.001
	Deceased	2	428	428	MD = 0.88	0.38, 1.38	0.0006	0	0.38	
POD7 urine output	Living	3	120	120	MD = -0.17	-0.54, 0.19	0.35	0	0.80	–
	Deceased	–	–	–	–	–	–	–	–	
Blood pH	Living	3	120	120	MD = 0.05	0.02, 0.069	0.004	87	0.0006	0.87
	Deceased	3	380	370	MD = 0.05	0.04, 0.06	<0.00001	19	0.29	
Bicarbonate levels	Living	3	120	120	MD = 2.72	0.42, 5.02	0.02	90	<0.0001	0.52
	Deceased	3	459	467	MD = 1.95	1.57, 2.34	<0.00001	0	0.43	
Base excess	Living	3	120	120	MD = 2.72	0.59, 4.86	0.01	92	<0.00001	0.36
	Deceased	1	42	43	MD = 1.40	-0.47, 3.27	0.14	–	–	
Serum chloride	Living	3	120	120	MD = -12.48	-18.92, -6.04	0.0001	98	<0.00001	0.04
	Deceased	3	458	463	MD = -5.47	-6.86, -4.08	<0.00001	56	0.10	
Serum sodium	Living	1	30	30	MD = -2.26	-3.99, -0.53	0.01	–	–	0.14
	Deceased	3	467	469	MD = -0.93	-1.39, -0.46	<0.0001	0	0.72	
Serum potassium	Living	2	90	90	MD = 0.06	-0.27, 0.40	0.71	84	0.01	0.39
	Deceased	3	467	468	MD = -0.12	-0.36, 0.12	0.34	46	0.16	

PL, plasma-lyte solution; NS, normal saline; OR, odd ratio; MD, mean difference; POD, postoperative days.

### 3.5. Secondary outcomes

All forest plots for secondary outcomes were presented in Supplementary material.

#### 3.5.1. Post-operative serum creatinine.

Five studies documented the creatinine levels obtained on the POD1, POD2, and POD7 [7–10,16]. A meta-analysis revealed that there was no significant difference in serum creatinine levels at POD 1 (MD: -0.08, 95% CI: -0.17 to 0.34, P =  0.53), POD 2 (MD: -0.01, 95% CI: -0.22 to 0.25, P =  0.90), and POD 7 (MD: -0.07, 95% CI: -0.28 to 0.14, P =  0.53) between the two groups (Table 2). Homogeneity was seen in the blood creatinine levels measured on postoperative day 1 (POD 1) (P =  0.86, I² =  0%), POD 2 (P =  0.71, I² =  0%), and POD 7 (P =  0.39, I² =  3%). The test for subgroup analysis based on type of donor was not significant for POD 1 (P =  0.70), POD 2 (P =  0.48) and POD 7 (P =  0.08) respectively (Table 3).

#### 3.5.2. Post-operative urine output.

Four studies documented urine output at POD1 [7,9,10,16], while five studies at POD2 [7–10,16] and three studies at POD7 [9,10,16]. The results of the meta-analysis indicated a significant difference in urine output between the two groups regarding POD1 (MD: -1.22, 95% CI: -3.39 to 0.96, P = 0.27). No differences in urine output were observed between the two groups on POD2 (MD: -0.11, 95% CI: -1.03 to 0.80, P =  0.81) or POD7 (MD: -0.17, 95% CI: -0.54 to 0.19, P =  0.35). Urine output results showed heterogeneity on POD1 (P <  0.00001, I² =  90%) and POD2 (P =  0.00009, I² =  79%). On POD7, urine output results were homogeneous (P =  0.80, I² =  0%) (Table 2).

Subgroup analysis by donor type indicated that urine volume of the PL group was lower than that of the NS group in terms of the POD1 in the LDKT (MD: -2.14, 95% CI: -3.78 to-0.50, P = 0.01). Conversely, there was no significant difference between the PL and NS groups in DDKT in terms of the POD1 (MD: 1.22, 95% CI: -3.39 to 0.96, P =  0.05). Regarding POD2, the urine volume in the PL group from LDKT was significantly lower than that in the NS group (MD: -0.87, 95% CI: -1.74 to 0.08, P =  0.07), whereas the urine volume in the PL group from DDKT was marginally higher than that in the NS group (MD: 0.88, 95% CI: 0.38 to 1.38, P =  0.00006) (Table 3).

#### 3.5.3. Acid–base parameters (blood pH, bicarbonate, base excess).

Six studies documented the blood pH and bicarbonate levels immediately following surgery [7–10,15,16], while five studies recorded the levels of base excess [9,10,15,16]. Our meta-analysis demonstrated that compared to the NS group, the PL group had significantly higher immediate postoperative blood pH levels (MD: 0.05, 95% CI: 0.03 to 0.07, P <  0.00001), bicarbonate levels (MD: 2.09, 95% CI: 1.21 to 2.97, P <  0.00001), and base excess (MD: 2.42, 95% CI: 0.72 to 4.11, P = 0.005). The results indicated heterogeneity in blood pH (P =  0.003, I² =  72%), bicarbonate levels (P =  0.0003, I² =  78%), and base excess (P <  0.001, I² =  88%) (Table 2).

In LDKT subgroup analysis, pH and bicarbonate levels in the PL group were elevated compared to those in the NS group following living and deceased KT. No significant changes were noted for PH (P =  0.56) and bicarbonate (P =  0.16). In DDKT subgroup analysis, there was no significant difference in base excess between the two groups (MD: 1.40, 95% CI: -0.47 to 3.27, P =  0.14), and the base excess in the PL group was substantially greater than that in the NS group (MD: 1.81, 95% CI: 1.32-2.31, P <  0.00001) (Table 3).

#### 3.5.4. Serum electrolytes (chloride, sodium, potassium).

Six studies documented the serum chloride immediately following surgery [7–10,15,16], while four studies recorded the sodium [7–9,15] and five studies recorded the levels of potassium [7,8,10,15,16]. Our results demonstrated that compared to the NS group, the PL group had significantly higher immediate postoperative serum chloride (MD: -8.87, 95% CI: -13.50 to-4.25, P =  0.0002) and sodium levels (MD: -1.01, 95% CI: -1.46 to -0.57, P < 0.00001). However, there was no difference in postoperative potassium levels (MD: -0.03, 95% CI: -0.20 to 0.14, P =  0.73) between the two groups (Table 2). The results indicated homogeneity in postoperative sodium levels (P =  0.43, I² =  0%), but heterogeneity in the incidence of serum chloride (P <  0.00001, I² =  98%) and potassium (P <  0.03, I² =  64%) (Table 2).

Subgroup analysis based on donor type showed that the PL group had a consistent positive effect on postoperative serum chloride and sodium levels in both LDKT and DDKT subgroup analysis. No notable variation in postoperative potassium levels was seen in the subgroup analysis based on donor type, the difference was not statistically significant (P =  0.39) (Table 3).

#### 3.5.5. Publication bias.

**Fig 4:**
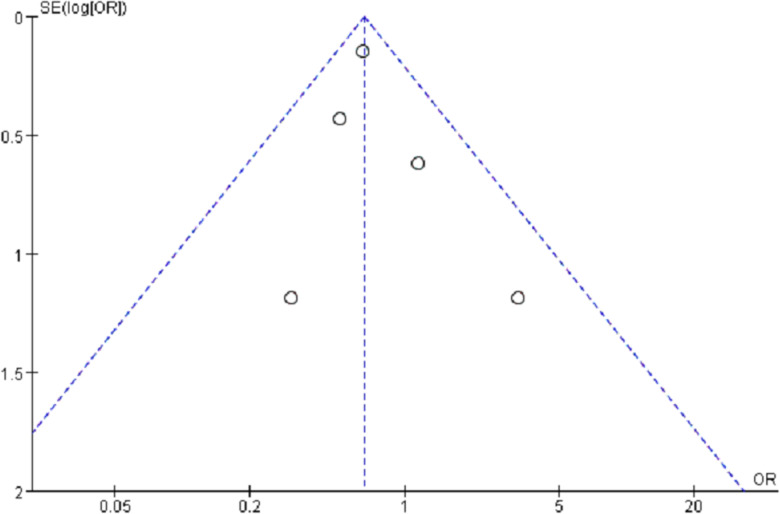
Funnel plot for DGF.

An assessment of publication bias concerning the DGF was performed utilizing a funnel plot (Fig 4). The bilateral symmetric funnel plot of the DGF did not indicate any substantial indication of publication bias.

## 4. Discussion

Our meta-analysis demonstrated that the PL group showed a significant advantage over the NS group in reducing the incidence of DGF. Acid-base parameters, including blood pH, bicarbonate, and base excess, were significantly higher in the PL group than in the NS group. Serum chloride and potassium concentrations were notably lower in the PL group compared to the NS group. Nonetheless, creatinine levels and serum potassium remained stable at postoperative days (POD) 1, 2, and 7.

Normal saline (NS) is extensively utilized in therapeutic settings. However, its elevated chloride concentration (154 mmol/L), exceeding physiological levels, may lead to hyperchloremic metabolic acidosis, potentially causing reduced renal perfusion and significant kidney injury [17–19]. An ideal crystalloid resuscitation solution should closely mimic the electrolyte composition of plasma. Lactated Ringer’s solution exhibits relatively low osmolality, in contrast to 0.9% saline, which may induce significant adverse effects due to its high chloride concentration. Unlike sodium chloride-based fluids, PL is a balanced salt solution with a similar electrolyte composition to plasma and does not produce the same disruption of the acid-base balance [20,21]. This phenomenon is explained by the presence of weak acids. At pH 7.4, the acids are predominantly present in their base anion forms, namely lactate, acetate, and gluconate. Acetate and gluconate serve as precursors to bicarbonate, with their primary metabolism occurring in the liver. However, it is possible for acetate to be converted into bicarbonate in other tissues, contributing to a reduced occurrence of acidosis. Moreover, the decreased chloride concentration in Plasma-lyte helps to suppress the decrease in the significant ion difference as compared to saline infusion [22,23]. The injection of NS exacerbated metabolic acidosis by substantially decreasing pH, blood ethanol concentration (BE), and serum iodine density (SID) throughout the postreperfusion period in living donor kidney transplantation. Patients administered NS exhibited hyperchloremic metabolic acidosis instead of dilutional hypochloremia [9]. When administered intraoperatively in renal transplant patients, PL is linked to superior pH and chloride levels in comparison to regular saline. Additionally, the potassium kinetics demonstrate safety, therefore confirming that PL can be safely utilized at increasing concentrations after renal transplantation [10].

In pathological studies, DGF is characterized by acute tubular necrosis (ATN). Renal ischemia and reperfusion injury following hypothermic preservation are critical pathogenic factors in its development. DGF is a key indicator of early graft function and a predictor of long-term graft survival [24]. The reduction in DGF observed with PL suggests that balanced crystalloids better support the kidney’s immediate post-transplant physiological needs, likely due to their physiological electrolyte composition and reduced risk of hyperchloremic metabolic acidosis [25]. Subgroup analysis by donor type showed no statistical significance, likely due to imbalances in the distribution of studies across subgroups. All three studies on LDKT found no association between PL and DGF improvement. However, PL significantly improved DGF outcomes in DDKT. A plausible explanation is that living donors provide superior graft functionality compared to deceased donors, enabling patients to better tolerate the adverse effects of NS. Further studies are needed to determine whether PL offers greater benefits for DGF in the context of LDKT.

No significant differences in serum creatinine levels were observed between the PL and NS groups at POD 1, 2, and 7. This indicated that, despite the reduced incidence of DGF with PL, the overall kidney function as measured by serum creatinine did not differ significantly between the groups in the immediate postoperative period [26]. A significant increase in urine output on POD 1 was observed in the PL group, suggesting improved immediate graft function. The initial higher urine output could be attributed to better perfusion and less acidosis with PL, which may facilitate early diuresis [27]. The heterogeneity observed on POD 1 and 2 suggests variability in individual responses, likely influenced by factors such as the patient’s baseline kidney function and the nature of the transplant surgery. PL was associated with significantly better acid-base parameters immediately post-surgery, including higher blood pH, bicarbonate levels, and base excess [28]. This supports the hypothesis that balanced crystalloids like PL mitigate the acid-base disturbances commonly induced by NS due to its high chloride content [29]. The preservation of a more normal acid-base balance with PL could contribute to better overall metabolic stability in the immediate postoperative period, potentially reducing the risk of acidosis-related complications [25]. The use of PL led to significantly lower postoperative serum chloride and sodium levels than those observed with NS [26]. These findings align with the physiological composition of PL, which is designed to resemble plasma electrolytes more closely [30]. The lower chloride levels with PL reduce the risk of hyperchloremic acidosis, a known issue with NS [31]. Comparable postoperative potassium levels between the two groups indicate that PL does not pose additional risks of hyperkalemia, which is particularly important due to the risk of potassium imbalances in renal transplant patients [32].

DGF is a common and increasing problem in deceased donor kidney transplantation, driven by greater use of kidneys from older, increasingly multimorbid donors and DCD kidneys [33]. Intravenous fluid therapy is an inexpensive, yet critical part of the treatment given to all recipients of kidney transplantations. Because of the substantial morbidity and costs associated with DGF and subsequent need for dialysis, our finding of a substantial benefit for plasma-Lyte solution over saline without any concerning signals for harm provides a strong justification for a change to clinical practice. Kidney transplantation is practiced in countries across the full spectrum of income status [34]. The low cost and wide availability of balanced crystalloids like plasma-Lyte solution make this intervention readily implementable at transplantation centers globally.

### 4.1. Strength and limitations

Previous meta-analyses have been conducted to explore the optimal intravenous fluid for KT [35–39]. The meta-analysis performed by Imran et al. [39] compared balanced crystal solution (BC) and NS. Specially, our study was the first meta-analysis to compare the efficacy and safety of PL and NS for KT. However, we recognize the potential limitations of our work. Initially, just six RCTs were included due to our stringent inclusion requirements. The statistical results of partial clinical outcomes were insufficient to demonstrate the difference between the two groups due to the limited sample size. Secondly, the study includes inadequate power to compare the effects of NS and PL on short- or long-term graft function and other postoperative complications; addressing this will require a larger, multicenter study. Furthermore, we were unable to manage confounding variables such as varying inclusion criteria, population disparities, and the differing levels of surgeon ability, which may lead to study heterogeneity and bias. Consequently, more clinical outcomes reported by randomized controlled trials are essential to further validate the benefits of PL.

### 4.2. Implications for future practice

The systematic review and meta-analysis comparing PL versus NS during KT highlight several critical insights and implications for clinical practice and future research in the field of kidney transplantation. One of the most significant findings from the meta-analysis is the reduced risk of DGF associated with the use of PL compared to NS. This reduction in DGF is crucial because DGF is a key predictor of early graft function and long-term graft survival [40]. The more physiological electrolyte composition of PL, which reduces the risk of hyperchloremic metabolic acidosis, appears to support the immediate post-transplant needs of the kidney more effectively than NS [38]. This suggests that incorporating PL into standard intraoperative fluid management protocols could enhance early graft function, potentially improving patient outcomes and graft longevity [32]. While the immediate postoperative benefits of PL are evident, long-term studies are needed to assess whether these initial advantages translate into sustained improvements in graft function and survival [41]. Future research should also explore the cost-effectiveness of using PL over NS, given the widespread use of intravenous fluids in clinical practice [42]. If PL proves to be more cost-effective by reducing the incidence of DGF and related complications, it could justify its broader adoption in transplant surgery protocols [43]. The variability in individual responses to PL and NS, as seen in urine output heterogeneity on POD 1 and 2, suggests that personalized fluid management strategies could be beneficial [32]. Future research should aim to identify specific patient populations that might benefit more from PL based on factors such as baseline kidney function, type of donor (living vs. deceased), and other comorbidities. Tailoring fluid management to individual patient needs could optimize outcomes further. One of the limitations of our study includes the lack of a long-term follow-up in assessing graft survival. We did not prospectively assess 30-day mortality, although retrospectively all patients, in all groups, did arrive for scheduled follow-up visits even at 90 days post-transplant. This possibly means in our cohort the type of perioperative intravenous fluid did not significantly influence mortality. Assessing a biomarker such as neutrophil gelatinase-associated lipocalin (NGAL) or serum renalase either as part of the study or in the follow-up clinic may have given some additional information [10].

## 5. Conclusion

In summary, this meta-analysis indicated that using PL after kidney transplantation is linked to a decreased likelihood of DGF and improved acid-base balance, without any negative impact on potassium levels, when compared to NS. The results of this study provide evidence in favor of prioritizing the use of PL in perioperative care to enhance transplant outcomes and increase graft survival. This makes a strong argument for its use in clinical practice. Nevertheless, further research is necessary to validate these enduring advantages and to enhance fluid-management techniques for various patient groups receiving kidney transplantation.

## Supporting information

S1 PRIMA Checklist PRIMA checklist. (DOCX)

S1 Material Methodological details and additional figures. (DOCX)
