# Supplementary material for: Plasma-lyte solution versus saline in kidney transplantation: A systematic review and meta-analysis of randomized controlled trials
Source: PLoS One. 2025 Apr 9;20(4):e0320082. doi: 10.1371/journal.pone.0320082 (PMC11981191; doi:10.1371/journal.pone.0320082)
Supplement: S1 Material — (DOCX) [file pone.0320082.s002.docx]

**Supplement material for**

**Plasma-Lyte solution versus saline in kidney trans-plantation: a systematic review and meta-analysis of randomized controlled trials**

Yucai Chang^1†^, Yuechen Qin^1†^, Yue Zou^1^, Haijian Zeng^1^, Chunlan Li^1^, Mengtian Qin^1^, Jianyu Wu^1^, Jian Ban^1*^

^1^ The First Affiliated Hospital of Guangxi University of Science and Technology, Guangxi University of Science and Technology, 124 Yuejin Road, Liuzhou, Guangxi Province 545001, China

**Content**

[**Tables** 2](#_Toc190858303)

[S1 Table: The details of the searching record in PubMed 2](#_Toc190858304)

[S2 Table: The details of the searching record in Cochrane library 3](#_Toc190858305)

[S3 Table: The details of the searching record in Embase 4](#_Toc190858306)

[S4 Table: The details of the searching record in Web of science 5](#_Toc190858307)

[S5 Table: A numbered table of all studies 6](#_Toc190858308)

[S6 Table: Confirmation that the study was eligible to be included in the review. 20](#_Toc190858309)

[S7 Table: Data extraction 21](#_Toc190858310)

[**Figures** 25](#_Toc190858311)

[S1 Fig: Forest plot of subgroup according to the type of donor for DGF 25](#_Toc190858312)

[S2 Fig: : Forest plot of subgroup according to the type of donor 25](#_Toc190858313)

[S2 Fig A: Forest plot of subgroup according to the type of donor for POD1 serum creatinine 25](#_Toc190858314)

[S2 Fig B: Forest plot of subgroup according to the type of donor for POD2 serum creatinine 26](#_Toc190858315)

[S2 Fig C: Forest plot of subgroup according to the type of donor for POD7 serum creatinine 26](#_Toc190858316)

[S3 Fig: Forest plot of subgroup according to the type of donor 27](#_Toc190858317)

[S3 Fig A: Forest plot of subgroup according to the type of donor for POD1 urine output 27](#_Toc190858318)

[S3 Fig B: Forest plot of subgroup according to the type of donor for POD2 urine output 27](#_Toc190858319)

[S3 Fig C: Forest plot of subgroup according to the type of donor for POD7 urine output 27](#_Toc190858320)

[S4 Fig: Forest plot of subgroup according to the type of donor for Blood pH 28](#_Toc190858321)

[S5 Fig: Forest plot of subgroup according to the type of donor for Bicarbonate levels 28](#_Toc190858322)

[S6 Fig: Forest plot of subgroup according to the type of donor for Base excess 28](#_Toc190858323)

[S7 Fig: Forest plot of subgroup according to the type of donor for Serum chloride 29](#_Toc190858324)

[S8 Fig: Forest plot of subgroup according to the type of donor for Serum sodium 29](#_Toc190858325)

[S9 Fig: Forest plot of subgroup according to the type of donor for Serum potassium 29](#_Toc190858326)

# Tables

## S1 Table: The details of the searching record in PubMed

| PubMed | | |
| --- | --- | --- |
| No. | Query | Results |
| #1 | (((((((((kidney transplantation[Title/Abstract]) OR (Renal Transplantation[Title/Abstract])) OR (Renal Transplantations[Title/Abstract])) OR (Transplantations, Renal[Title/Abstract])) OR (Transplantation, Renal[Title/Abstract])) OR (Grafting, Kidney[Title/Abstract])) OR (Kidney Grafting[Title/Abstract])) OR (Transplantation, Kidney[Title/Abstract])) OR (Kidney Transplantations[Title/Abstract])) OR (Transplantations, Kidney[Title/Abstract]) | 60,758  Results (2024.8.2) |
| #2 | (((((Saline Solution[Title/Abstract]) OR (0.9% Saline[Title/Abstract])) OR (Saline, 0.9%[Title/Abstract])) OR (0.9% NaCl[Title/Abstract])) OR (Normal Saline[Title/Abstract])) OR (Saline, Normal[Title/Abstract]) | 51,936  Results (2024.8.2) |
| #3 | ((Plasma-Lyte 148) OR (plasma-lyte)) OR (Plasmalyte) | 155,427  Results (2024.8.2) |
| #4 | ((((("Prospective Studies"[Mesh]) OR (Prospective Study[Title/Abstract])) OR (Studies, Prospective[Title/Abstract])) OR (Study, Prospective[Title/Abstract])) OR (‘’Prospective Comparative Study’’’[Title/Abstract])) OR ( Prospective[Title/Abstract]) | 1,015,798  Results (2024.8.2) |
| #5 | ((("Retrospective Studies"[Mesh]) OR (Studies, Retrospective[Title/Abstract])) OR (Study, Retrospective[Title/Abstract])) OR (Retrospective Study[Title/Abstract]) | 1,278,894  Results (2024.8.2) |
| #6 | (randomized controlled trial [pt] OR controlled clinical trial [pt] OR randomized [tiab] OR placebo [tiab] OR clinical trials as topic [mesh:noexp] OR randomly [tiab] OR trial [ti]) NOT (animals [mh] NOT humans [mh]) | 1,503,276  Results (2024.8.2) |
| #7 | 1 AND 2 AND 3 | 33  Results (2024.8.2) |
| #8 | 4 OR 5 OR 6 | 3,488,639  Results (2024.8.2) |
| #9 | 7 AND 8 | 24  Results (2024.8.2) |

## S2 Table: The details of the searching record in Cochrane library

| Cochrane library | | |
| --- | --- | --- |
| No. | Query | Results |
| #1 | (kidney transplantation OR Renal Transplantation OR Renal Transplantations OR Transplantations, Renal OR Transplantation, Renal OR Grafting, Kidney OR Kidney Grafting OR Transplantation, Kidney):ab,ti,kw | 12590  Results (2024.8.2) |
| #2 | (Saline Solution OR 0.9% Saline OR 0.9% NaCl OR Saline, 0.9%  OR Normal Saline OR Saline, Normal):ab,ti,kw | 30421  Results (2024.8.2) |
| #3 | (plasma-lyte OR 0.9% Saline OR Plasmalyte):ab,ti,kw | 6334  Results (2024.8.2) |
| #4 | (Prospective Studies OR Prospective Study OR Studies, Prospective OR Study, Prospective OR Prospective Comparative Study OR Prospective):ab,ti,kw | 273374  Results (2024.8.2) |
| #5 | (randomized controlled trial OR controlled clinical trial OR randomized OR placebo OR clinical trials as topic OR randomly OR trial):ab,ti,kw | 1554809  Results (2024.8.2) |
| #6 | (Retrospective Studies OR Studies, Retrospective OR Study, Retrospective OR Retrospective Study):ab,ti,kw | 41128  Results (2024.8.2) |
| #7 | #1AND#2AND#3 | 63  Results (2024.8.2) |
| #8 | #4OR#5OR#6 | 1588257  Results (2024.8.2) |
| 9 | #7 AND #8 | 58  Results (2024.8.2) |

## S3 Table: The details of the searching record in Embase

| Embase | | |
| --- | --- | --- |
| No. | Query | Results |
| #1 | ‘kidney transplantation’/exp OR ‘Renal Transplantation’:ab,ti,kw OR ‘Renal Transplantations’:ab,ti,kw OR ‘Transplantations, Renal’:ab,ti,kw OR ‘Transplantation, Renal’:ab,ti,kw OR ‘Grafting, Kidney’:ab,ti,kw OR ‘Kidney Grafting’:ab,ti,kw OR ‘Transplantation, Kidney’:ab,ti,kw | 208,134  Results (2024.8.2) |
| #2 | 'Saline Solution'/exp OR '0.9% Saline' OR '0.9% NaCl':ab,ti,kw OR 'Normal Saline':ab,ti,kw OR 'Saline, Normal':ab,ti,kw OR 'Saline, 0.9%':ab,ti,kw | 279,031  Results (2024.8.2) |
| #3 | ' Plasma-Lyte 148'/exp OR ' plasma-lyte ' OR ' Plasmalyte ':ab,ti,kw | 1,239  Results (2024.8.2) |
| #4 | ‘Prospective Studies’:ab,ti,kw OR ‘Prospective Study’:ab,ti,kw OR ‘Studies, Prospective’:ab,ti,kw OR ‘Study, Prospective’:ab,ti,kw OR ‘Prospective Comparative Study’:ab,ti,kw OR ‘Prospective’:ab,ti,kw | 1,175,350  Results (2024.8.2) |
| #5 | ‘Retrospective Studies’:ab,ti,kw OR 'Studies, Retrospective’:ab,ti,kw OR ’Study, Retrospective’:ab,ti,kw OR ‘Retrospective Study’:ab,ti,kw | 393,288  Results (2024.8.2) |
| #6 | 'randomized controlled trial'/exp OR 'randomized controlled trial':ti,ab,it OR 'randomized':ti,ab,it OR 'randomised':ti,ab,it OR 'randomization':ti,ab,it OR 'randomisa- tion':ti,ab,it OR rct:ti,ab,it OR 'randomly':ti,ab,it OR pla-cebo:ti,ab,it | 1,833,005  Results (2024.8.2) |
| #7 | #1 AND #2 AND #3 | 40  Results (2024.8.2) |
| #8 | #4 OR #5 OR #6 | 3,143,723  Results (2024.8.2) |
| #9 | #7 AND #8 | 18  Results (2024.8.2) |

## S4 Table: The details of the searching record in Web of science

| Web of science | | |
| --- | --- | --- |
| No. | Query | Results |
| #1 | TS=(kidney transplantation OR Renal Transplantation OR Renal Transplantations OR Transplantations, Renal OR Transplantation, Renal OR Grafting, Kidney OR Kidney Grafting OR Transplantation, Kidney) | 273,830  Results (2024.8.2) |
| #2 | TS=(Saline Solution OR 0.9% Saline OR 0.9% NaCl OR Normal Saline OR Saline, Normal OR Saline, 0.9% ) | 193,950  Results (2024.8.2) |
| #3 | TS=( Plasma-Lyte OR plasma-lyte OR Plasmalyte ) | 611  Results (2024.8.2) |
| #4 | TS=(Prospective Studies OR Prospective Study OR Studies, Prospective OR Study, Prospective OR Prospective Comparative Study OR Prospective) | 1,359,465  Results (2024.8.2) |
| #5 | TS=(Retrospective Studies OR Studies, Retrospective OR Study, Retrospective OR Retrospective Study ) | 1,600,004  Results (2024.8.2) |
| #6 | TS=(randomized controlled trial OR controlled clinical trial OR randomized OR placebo OR clinical trials as topic OR randomly OR trial) | 4,447,905  Results (2024.8.2) |
| #7 | #1AND#2AND#3 | 28  Results (2024.8.2) |
| #8 | #4 OR #5 OR #6 | 6,795,115  Results (2024.8.2) |
| #9 | #7 AND #8 | 26  Results (2024.8.2) |

## S5 Table: A numbered table of all studies

| This work was performed by Yucai Chang and Yuechen Qin | Author | Year | Title | Journal | DOI |
| --- | --- | --- | --- | --- | --- |
| Included Studies (n=6) | Collins, M. G., et al. | 2023 | Balanced crystalloid solution versus saline in deceased donor kidney transplantation (BEST-Fluids): a pragmatic, double-blind, randomised, controlled trial | Lancet (london, england) | 10.1016/S0140-6736(23)00642-6 |
|  | do Nascimento Junior, P., et al. | 2022 | Effects of Plasma-Lyte.½ and 0.9% saline in renal function after deceased-donor kidney transplant: a randomized controlled trial | Brazilian journal of anesthesiology (english edition) | 10.1016/j.bjane.2021.08.015 |
|  | Hadimioglu, N., et al. | 2008 | The effect of different crystalloid solutions on acid-base balance and early kidney function after kidney transplantation | Anesthesia and Analgesia | 10.1213/ane.0b013e3181732d64 |
|  | Kim, S. Y., et al. | 2013 | Comparison of the effects of normal saline versus plasmalyte on acid-base balance during living donor kidney transplantation using the Stewart and base excess methods | Transplantation Proceedings | 10.1016/j.transproceed.2013.02.124 |
|  | Saini, V., et al. | 2021 | Normal Saline Versus Balanced Crystalloids in Renal Transplant Surgery: A Double-Blind Randomized Controlled Study | Cureus | 10.7759/cureus.18247 |
|  | Weinberg, L., et al. | 2017 | Effects of intraoperative and early postoperative normal saline or Plasma-Lyte 148 ® on hyperkalaemia in deceased donor renal transplantation: A double-blind randomized trial | British Journal of Anaesthesia | 10.1093/bja/aex163 |
| Duplicates remove(n=42) | Abdallah, E., et al. | 2014 | Comparison between the effects of intraoperative human albumin and normal saline on early graft function in renal transplantation | International urology and nephrology | 10.1007/s11255-014-0785-z |
|  | Bhaskaran, K., et al. | 2018 | A prospective, randomized, comparison study on effect of perioperative use of chloride liberal intravenous fluids versus chloride restricted intravenous fluids on postoperative acute kidney injury in patients undergoing off-pump coronary artery bypass grafting surgeries | Annals of cardiac anaesthesia | 10.4103/aca.ACA_230_17 |
|  | Carvalho Pereira, L., et al. | 2024 | Balanced Crystalloids Versus Normal Saline in Kidney Transplant Patients: An Updated Systematic Review, Meta-analysis, and Trial Sequential Analysis | Anesth Analg | 10.1213/ane.0000000000006932 |
|  | Collins, M. G., et al. | 2020 | Study Protocol for Better Evidence for Selecting Transplant Fluids (BEST-Fluids): a pragmatic, registry-based, multi-center, double-blind, randomized controlled trial evaluating the effect of intravenous fluid therapy with Plasma-Lyte 148 versus 0.9% saline on delayed graft function in deceased donor kidney transplantation | Trials | 10.1186/s13063-020-04359-2 |
|  | Collins, M. G., et al. | 2020 | Study Protocol for Better Evidence for Selecting Transplant Fluids (BEST-Fluids): A pragmatic, registry-based, multi-center, double-blind, randomized controlled trial evaluating the effect of intravenous fluid therapy with Plasma-Lyte 148 versus 0.9% saline on delayed graft function in deceased donor kidney transplantation | Trials | 10.1186/s13063-020-04359-2 |
|  | Collins, M. G., et al. | 2020 | Study Protocol for Better Evidence for Selecting Transplant Fluids (BEST-Fluids): a pragmatic, registry-based, multi-center, double-blind, randomized controlled trial evaluating the effect of intravenous fluid therapy with Plasma-Lyte 148 versus 0.9% saline on delayed graft function in deceased donor kidney transplantation | Trials | 10.1186/s13063-020-04359-2 |
|  | Collins, M. G., et al. | 2023 | Balanced crystalloid solution versus saline in deceased donor kidney transplantation (BEST-Fluids): a pragmatic, double-blind, randomised, controlled trial | Lancet | 10.1016/s0140-6736(23)00642-6 |
|  | Collins, M. G., et al. | 2023 | Balanced crystalloid solution versus saline in deceased donor kidney transplantation (BEST-Fluids): a pragmatic, double-blind, randomised, controlled trial | Lancet | 10.1016/s0140-6736(23)00642-6 |
|  | Collins, M. G., et al. | 2023 | Balanced crystalloid solution versus saline in deceased donor kidney transplantation (BEST-Fluids): a pragmatic, double-blind, randomised, controlled trial | The Lancet | 10.1016/S0140-6736(23)00642-6 |
|  | Collins, M. G., et al. | 2022 | Baseline Characteristics and Representativeness of Participants in the BEST- Fluids Trial: A Randomized Trial of Balanced Crystalloid Solution Versus Saline in Deceased Donor Kidney Transplantation | Transplantation Direct | 10.1097/txd.0000000000001399 |
|  | Collins, M. G., et al. | 2022 | Baseline Characteristics and Representativeness of Participants in the BEST-Fluids Trial: A Randomized Trial of Balanced Crystalloid Solution Versus Saline in Deceased Donor Kidney Transplantation | Transplantation Direct | 10.1097/txd.0000000000001399 |
|  | Collins, M. G., et al. | 2022 | Baseline Characteristics and Representativeness of Participants in the BEST-Fluids Trial: A Randomized Trial of Balanced Crystalloid Solution Versus Saline in Deceased Donor Kidney Transplantation | Transplantation direct | 10.1097/txd.0000000000001399 |
|  | Collins, M. G., et al. | 2022 | Baseline Characteristics and Representativeness of Participants in the BEST-Fluids Trial: A Randomized Trial of Balanced Crystalloid Solution Versus Saline in Deceased Donor Kidney Transplantation | Transplant Direct | 10.1097/txd.0000000000001399 |
|  | do Nascimento Junior, P., et al. | 2022 | Effects of Plasma-Lyte.½ and 0.9% saline in renal function after deceased-donor kidney transplant: a randomized controlled trial | Brazilian journal of anesthesiology | 10.1016/j.bjane.2021.08.015 |
|  | do Nascimento Junior, P., et al. | 2022 | Effects of Plasma-Lyte® and 0.9% saline in renal function after deceased-donor kidney transplant: a randomized controlled trial | Brazilian Journal of Anesthesiology | 10.1016/j.bjane.2021.08.015 |
|  | do Nascimento Junior, P., et al. | 2022 | Effects of Plasma-Lyte.½ and 0.9% saline in renal function after deceased-donor kidney transplant: a randomized controlled trial | Braz J Anesthesiol | 10.1016/j.bjane.2021.08.015 |
|  | do Nascimento Junior, P., et al. | 2022 | Effects of Plasma-Lyte.½ and 0.9% saline in renal function after deceased-donor kidney transplant: a randomized controlled trial | Brazilian Journal of Anesthesiology (English Edition) | 10.1016/j.bjane.2021.08.015 |
|  | Hadimioglu, N., et al. | 2008 | The effect of different crystalloid solutions on acid-base balance and early kidney function after kidney transplantation | Anesth Analg | 10.1213/ane.0b013e3181732d64 |
|  | Hadimioglu, N., et al. | 2008 | The effect of different crystalloid solutions on acid-base balance and early kidney function after kidney transplantation | Anesthesia and Analgesia | 10.1213/ane.0b013e3181732d64 |
|  | Hadimioglu, N., et al. | 2008 | The effect of different crystalloid solutions on acid-base balance and early kidney function after kidney transplantation | Anesthesia and analgesia | 10.1213/ane.0b013e3181732d64 |
|  | Harris, L. E., et al. | 2015 | The Effects of Normal Saline and an Acetate-Buffered Crystalloid Solution on Hyperkalemia in Deceased Donor Renal Transplantation: a Randomized Blinded Trial | Journal of the American Society of Nephrology : JASN | NA |
|  | Hayes, W. N., et al. | 2024 | A pragmatic, open-label, randomized controlled trial of Plasma-Lyte-148 versus standard intravenous fluids in children receiving kidney transplants (PLUTO) | Kidney International | 10.1016/j.kint.2023.09.032 |
|  | Huang, L., et al. | 2018 | Balanced crystalloids vs 0.9% saline for adult patients undergoing non-renal surgery: A meta-analysis | International Journal of Surgery | 10.1016/j.ijsu.2018.01.003 |
|  | Hurlburt, L., et al. | 2012 | Fluid management in deceased donor renal transplantation | Canadian journal of anesthesia | 10.1007/s12630-012-9785-6 |
|  | Kim, S. Y., et al. | 2013 | Comparison of the effects of normal saline versus Plasmalyte on acid-base balance during living donor kidney transplantation using the Stewart and base excess methods | Transplant Proc | 10.1016/j.transproceed.2013.02.124 |
|  | Kim, S. Y., et al. | 2013 | Comparison of the Effects of Normal Saline Versus Plasmalyte on Acid-Base Balance During Living Donor Kidney Transplantation Using the Stewart and Base Excess Methods | Transplantation Proceedings | 10.1016/j.transproceed.2013.02.124 |
|  | Kim, S. Y., et al. | 2013 | Comparison of the effects of normal saline versus Plasmalyte on acid-base balance during living donor kidney transplantation using the Stewart and base excess methods | Transplantation proceedings | 10.1016/j.transproceed.2013.02.124 |
|  | Medeiros, H., et al. | 2023 | A Comparison Between Saline and Balanced Solutions in Kidney Transplants: a Randomized Clinical Trial | Cureus | 10.7759/cureus.49813 |
|  | Medeiros, H., et al. | 2023 | A Comparison Between Saline and Balanced Solutions in Kidney Transplants: A Randomized Clinical Trial | Cureus | 10.7759/cureus.49813 |
|  | O'Malley, C. M. N., et al. | 2005 | A randomized, double-blind comparison of lactated Ringer's solution and 0.9% NaCl during renal transplantation | Anesthesia and analgesia | 10.1213/01.ANE.0000150939.28904.81 |
|  | Park, J. H., et al. | 2022 | Effects of intraoperative dexmedetomidine infusion on renal function in elective living donor kidney transplantation: a randomized controlled trial | Journal canadien d'anesthesie [Canadian journal of anaesthesia] | 10.1007/s12630-021-02173-1 |
|  | Pascoe, E. M., et al. | 2022 | Statistical analysis plan for Better Evidence for Selecting Transplant Fluids (BEST-Fluids): a randomised controlled trial of the effect of intravenous fluid therapy with balanced crystalloid versus saline on the incidence of delayed graft function in deceased donor kidney transplantation | Trials | 10.1186/s13063-021-05989-w |
|  | Pascoe, E. M., et al. | 2022 | Statistical analysis plan for Better Evidence for Selecting Transplant Fluids (BEST-Fluids): a randomised controlled trial of the effect of intravenous fluid therapy with balanced crystalloid versus saline on the incidence of delayed graft function in deceased donor kidney transplantation | Trials | 10.1186/s13063-021-05989-w |
|  | Pascoe, E. M., et al. | 2022 | Statistical analysis plan for Better Evidence for Selecting Transplant Fluids (BEST-Fluids): a randomised controlled trial of the effect of intravenous fluid therapy with balanced crystalloid versus saline on the incidence of delayed graft function in deceased donor kidney transplantation | Trials | 10.1186/s13063-021-05989-w |
|  | Potura, E., et al. | 2015 | An acetate-buffered balanced crystalloid versus 0.9% saline in patients with end-stage renal disease undergoing cadaveric renal transplantation: a prospective randomized controlled trial | Anesthesia and analgesia | 10.1213/ANE.0000000000000419 |
|  | Saini, V., et al. | 2021 | Normal Saline Versus Balanced Crystalloids in Renal Transplant Surgery: A Double-Blind Randomized Controlled Study | Cureus Journal of Medical Science | 10.7759/cureus.18247 |
|  | Saini, V., et al. | 2021 | Normal Saline Versus Balanced Crystalloids in Renal Transplant Surgery: a Double-Blind Randomized Controlled Study | Cureus | 10.7759/cureus.18247 |
|  | Shah, R. B., et al. | 2014 | Effect of intraoperative human albumin on early graft function in renal transplantation | Saudi journal of kidney diseases and transplantation | 10.4103/1319-2442.144246 |
|  | Weinberg, L., et al. | 2017 | Effects of intraoperative and early postoperative normal saline or Plasma-Lyte 148® on hyperkalaemia in deceased donor renal transplantation: a double-blind randomized trial | Br J Anaesth | 10.1093/bja/aex163 |
|  | Weinberg, L., et al. | 2017 | Effects of intraoperative and early postoperative normal saline or Plasma-Lyte 148® on hyperkalaemia in deceased donor renal transplantation: a double-blind randomized trial | British Journal of Anaesthesia | 10.1093/bja/aex163 |
|  | Weinberg, L., et al. | 2017 | Effects of intraoperative and early postoperative normal saline or Plasma-Lyte 148® on hyperkalaemia in deceased donor renal transplantation: a double-blind randomized trial | British journal of anaesthesia | 10.1093/bja/aex163 |
|  | Weinberg, L., et al. | 2017 | Effects of intraoperative and early postoperative normal saline or Plasma-Lyte 148® on hyperkalaemia in deceased donor renal transplantation: a double-blind randomized trial | British journal of anaesthesia | 10.1093/bja/aex163 |
| Other type of articles(n=36) | Abdallah, E., et al. | 2017 | Comparison between the effects of intraoperrative human albumin and normal saline on earrly graft function in kidney transplantation | Nephrology dialysis transplantation | 10.1093/ndt/gfx182 |
|  | Actrn | 2017 | An investigator-initiated, pragmatic, registry-based, multi-centre, double-blind, randomised controlled trial evaluating the effect of Plasmalyte versus 0.9% saline on early kidney transplant function in deceased donor kidney transplantation | https://trialsearch.who.int/Trial2.aspx?TrialID=ACTRN12617000358347 | NA |
|  | Bateman, R. M., et al. | 2016 | 36th International Symposium on Intensive Care and Emergency Medicine : Brussels, Belgium. 15-18 March 2016 | Crit Care | 10.1186/s13054-016-1208-6 |
|  | Carvalho Pereira, L., et al. | 2024 | Balanced Crystalloids Versus Normal Saline in Kidney Transplant Patients: An Updated Systematic Review, Meta-analysis, and Trial Sequential Analysis | Anesthesia and Analgesia | 10.1213/ANE.0000000000006932 |
|  | ChiCtr | 2019 | Effect of ulinastatin on prevention of acute kidney injury after cardiac surgery: a randomised, double-blinded, placebo-controlled trial | https://trialsearch.who.int/Trial2.aspx?TrialID=ChiCTR1900026747 | NA |
|  | Collins, M. G., et al. | 2022 | Baseline Characteristics and Representativeness of the BEST-Fluids Trial Participants: a Randomized Trial of Balanced Crystalloid Solution vs. Saline in Deceased Donor Kidney Transplantation | Journal of the American Society of Nephrology : JASN | NA |
|  | Collins, M. G., et al. | 2022 | Baseline Characteristics and Representativeness of the BEST-Fluids Trial Participants: A Randomized Trial of Balanced Crystalloid Solution vs. Saline in Deceased Donor Kidney Transplantation | Journal of the American Society of Nephrology | NA |
|  | fzq2w, R. B. R. | 2023 | Evaluation of the Types of Hydration Solutions Used in Renal Transplantation | https://trialsearch.who.int/Trial2.aspx?TrialID=RBR-5fzq2w7 | NA |
|  | Gulyam Kuruba, S. M., et al. | 2011 | Unilateral transversus abdominis plane block for renal transplant recipients | British journal of anaesthesia | 10.1093/bja/aer234 |
|  | Harris, L. E., et al. | 2015 | The Effects of Normal Saline and an Acetate-Buffered Crystalloid Solution on Hyperkalemia in Deceased Donor Renal Transplantation: A Randomized Blinded Trial | Journal of the American Society of Nephrology | NA |
|  | Hayes, W., et al. | 2023 | PLASMA-LYTE-148 VERSUS STANDARD INTRAVENOUS FLUID IN CHILDREN RECEIVING KIDNEY TRANSPLANTS (PLUTO): A PRAGMATIC, OPEN-LABEL, RANDOMISED CONTROLLED TRIAL | Pediatric Nephrology | 10.1007/s00467-023-06094-7 |
|  | Huang, L., et al. | 2018 | Balanced crystalloids vs 0.9% saline for adult patients undergoing non-renal surgery: A meta-analysis | Int J Surg | 10.1016/j.ijsu.2018.01.003 |
|  | Hurlburt, L., et al. | 2012 | Fluid management in deceased donor renal transplantation | Canadian Journal of Anesthesia | 10.1007/s12630-012-9785-6 |
|  | Imran, M., et al. | 2024 | Perioperative balanced crystalloids versus normal saline during kidney transplantation: a systematic review and meta-analysis of randomized controlled trials | Int Urol Nephrol | 10.1007/s11255-023-03936-z |
|  | Isrctn | 2019 | Propofol in cardiac surgery: proMPT-2 | https://trialsearch.who.int/Trial2.aspx?TrialID=ISRCTN15255199 | NA |
|  | Jahangir, A., et al. | 2021 | Comparison of normal saline solution with low-chloride solutions in renal transplants: a meta-analysis | Kidney Res Clin Pract | 10.23876/j.krcp.21.027 |
|  | Kalal, C. R., et al. | 2016 | Hypertonic saline versus mannitol in the management of cerebral edema in acute liver failure: a randomized open label control trial | Journal of hepatology | NA |
|  | Murphy, C., et al. | 2012 | Transversus abdominis plane block in cadaveric renal transplantation: a randomized trial | Canadian journal of anesthesia | 10.1007/s12630-012-9785-6 |
|  | Nct | 2008 | Busulfan Plus Cyclophosphamide vs Fludarabine as a Conditioning Regimen | https://clinicaltrials.gov/show/NCT00774280 | NA |
|  | Nct | 2010 | Perioperative Fluid Management in Patients Receiving Cadaveric Renal Transplants | https://clinicaltrials.gov/show/NCT01075750 | NA |
|  | Nct | 2013 | Safety and Efficacy Study of Intravenous Immunoglobulin to Treat Japanese Encephalitis | https://clinicaltrials.gov/show/NCT01856205 | NA |
|  | Nct | 2015 | Perioperative Fluid Management in Patients Receiving Major Abdominal Surgery - Effects of Normal Saline Versus an Acetate Buffered Balanced Infusion Solution on the Necessity of Catecholamines for Cardiocirculatory Support | https://clinicaltrials.gov/show/NCT02414555 | NA |
|  | Nct | 2017 | Efficacy Transverse Abdominal Plane (TAP) Block Renal Transplant Surgery | https://clinicaltrials.gov/show/NCT03181438 | NA |
|  | Nct | 2018 | Dexmedetomidine and Liver Transplantation | https://clinicaltrials.gov/show/NCT03770130 | NA |
|  | Nct | 2018 | Effects of Faecal Microbiota Transplantation in Patients With IBS | https://clinicaltrials.gov/show/NCT03822299 | NA |
|  | Nct | 2019 | Better Evidence for Selecting Transplant Fluids | https://clinicaltrials.gov/show/NCT03829488 | NA |
|  | Nct | 2020 | Effects of Fecal Microbiota Transplantation on Weight in Obese Patients With Non-alcoholic Fatty Liver Disease | https://clinicaltrials.gov/show/NCT04594954 | NA |
|  | Nct | 2021 | Fecal Microbiota Transplantation and ACHIM for Manipulating Gut Microbiota in IBS Patients | https://clinicaltrials.gov/ct2/show/NCT05088434 | NA |
|  | Nct | 2021 | Mannitol Use During Cadaveric Kidney Transplantation | https://clinicaltrials.gov/show/NCT04705402 | NA |
|  | Nct | 2023 | Hypertonic Saline Solution to Prevent Acute Kidney Injury After Heart Transplantation | https://clinicaltrials.gov/show/NCT05909150 | NA |
|  | p888f, R. B. R. | 2023 | Preventive correction of fibrinolysis with Epsilon Aminocaproic Acid detected by Thromboelastometry during Liver Transplant | https://trialsearch.who.int/Trial2.aspx?TrialID=RBR-105p888f | NA |
|  | Rajput, P., et al. | 2017 | Donor blood group plasma sans plasmapharesis in ABO incompatible transplant-an in vitro and in vivo study | Indian Journal of Nephrology | NA |
|  | Ritschl, P., et al. | 2016 | Peri-operative organ perfusion with ATG-fresenius results in improved graft function in clinical liver and kidney transplantation | Transplantation | 10.1097/01.tp.0000490147.72544.1a |
|  | Vignarajah, M., et al. | 2023 | Comparing the Intraoperative use of Balanced Crystalloids Vs. 0.9% Saline on Postoperative Outcomes: A Systematic Review and Meta-Analysis | Canadian Journal of Anesthesia | 10.1007/s12630-023-02445-y |
|  | Wan, S., et al. | 2016 | Normal saline versus lower-chloride solutions for kidney transplantation | Cochrane Database of Systematic Reviews | 10.1002/14651858.CD010741.pub2 |
|  | Xue, M., et al. | 2019 | Low-chloride versus high-chloride crystalloid fluid on outcomes among critically ill adult patients | Critical Care Medicine | NA |
| Not relevant(n=34) | Abdallah, E., et al. | 2014 | Comparison between the effects of intraoperative human albumin and normal saline on early graft function in renal transplantation | Int Urol Nephrol | 10.1007/s11255-014-0785-z |
|  | Aydin, Z., et al. | 2012 | Randomized trial of short-course high-dose erythropoietin in donation after cardiac death kidney transplant recipients | American journal of transplantation | 10.1111/j.1600-6143.2012.04019.x |
|  | Barańska-Kosakowska, A., et al. | 2007 | Role of N-acetylcysteine on renal function in patients after orthotopic heart transplantation undergoing coronary angiography | Transplantation proceedings | 10.1016/j.transproceed.2007.08.057 |
|  | Bhaskaran, K., et al. | 2018 | A prospective, randomized, comparison study on effect of perioperative use of chloride liberal intravenous fluids versus chloride restricted intravenous fluids on postoperative acute kidney injury in patients undergoing off-pump coronary artery bypass grafting surgeries | Annals of cardiac anaesthesia | 10.4103/aca.ACA_230_17 |
|  | Blumberg, N., et al. | 2018 | 0.9% NaCl (Normal Saline) - Perhaps not so normal after all? | Transfusion and Apheresis Science | 10.1016/j.transci.2018.02.021 |
|  | Boer, C., et al. | 2018 | Choice of fluid type: physiological concepts and perioperative indications | British Journal of Anaesthesia | 10.1016/j.bja.2017.10.022 |
|  | Boldt, J., et al. | 2009 | Cardiopulmonary bypass priming using a high dose of a balanced hydroxyethyl starch versus an albumin-based priming strategy | Anesthesia and analgesia | 10.1213/ANE.0b013e3181b5a24b |
|  | Dey, A., et al. | 2018 | Comparison of normal saline and balanced crystalloid (plasmalyte) in patients undergoing elective craniotomy for supratentorial brain tumors: A randomized controlled trial | Neurology India | 10.4103/0028-3886.241347 |
|  | Forouzannia, S. K., et al. | 2013 | Adenosine preconditioning versus ischemic preconditioning in patients undergoing off-pump coronary artery bypass (OPCAB) | Journal of tehran university heart center | NA |
|  | Freir, N. M., et al. | 2012 | Transversus abdominis plane block for analgesia in renal transplantation: a randomized controlled trial | Anesthesia and analgesia | 10.1213/ANE.0b013e3182642117 |
|  | González-Castro, A., et al. | 2018 | Influence of Proportion of Normal Saline Administered in the Perioperative Period of Renal Transplantation on Kalemia Levels | Transplant Proc | 10.1016/j.transproceed.2017.06.040 |
|  | Goyal, V., et al. | 2021 | A randomized comparison between pulse pressure variation and central venous pressure in patients undergoing renal transplantation | Journal of Anaesthesiology Clinical Pharmacology | 10.4103/joacp.JOACP_23_20 |
|  | Gulyam Kuruba, S. M., et al. | 2014 | A randomised controlled trial of ultrasound-guided transversus abdominis plane block for renal transplantation | Anaesthesia | 10.1111/anae.12704 |
|  | Haines, R. W., et al. | 2019 | Managing Chloride and Bicarbonate in the Prevention and Treatment of Acute Kidney Injury | Seminars in Nephrology | 10.1016/j.semnephrol.2019.06.007 |
|  | Kainz, A., et al. | 2010 | Steroid pretreatment of organ donors to prevent postischemic renal allograft failure: a randomized, controlled trial | Annals of internal medicine | 10.7326/0003-4819-153-4-201008170-00003 |
|  | Khajavi, M. R., et al. | 2008 | Effects of normal saline vs. lactated ringer's during renal transplantation | Ren Fail | 10.1080/08860220802064770 |
|  | Langer, T., et al. | 2015 | Intravenous balanced solutions: from physiology to clinical evidence | Anaesthesiology intensive therapy | 10.5603/AIT.a2015.0079 |
|  | Medeiros, H., et al. | 2023 | A Comparison Between Saline and Balanced Solutions in Kidney Transplants: A Randomized Clinical Trial | Cureus Journal of Medical Science | 10.7759/cureus.49813 |
|  | Modi, M. P., et al. | 2012 | A comparative study of impact of infusion of Ringer's Lactate solution versus normal saline on acid-base balance and serum electrolytes during live related renal transplantation | Saudi J Kidney Dis Transpl | NA |
|  | Moreso, F., et al. | 2018 | Treatment of chronic antibody mediated rejection with intravenous immunoglobulins and rituximab: a multicenter, prospective, randomized, double-blind clinical trial | American journal of transplantation | 10.1111/ajt.14520 |
|  | Ni, C., et al. | 2020 | Study design of the DAS-OLT trial: a randomized controlled trial to evaluate the impact of dexmedetomidine on early allograft dysfunction following liver transplantation | Trials | 10.1186/s13063-020-04497-7 |
|  | O'Malley, C. M. N., et al. | 2005 | A randomized, double-blind comparison of lactated Ringer's solution and 0.9% NaCl during renal transplantation | Anesth Analg | 10.1213/01.Ane.0000150939.28904.81 |
|  | Othman, M. M., et al. | 2010 | The impact of timing of maximal crystalloid hydration on early graft function during kidney transplantation | Anesthesia and analgesia | 10.1213/ANE.0b013e3181d82ca8 |
|  | Park, J. H., et al. | 2022 | Effects of intraoperative dexmedetomidine infusion on renal function in elective living donor kidney transplantation: a randomized controlled trial | Journal canadien d'anesthesie [Canadian journal of anaesthesia] | 10.1007/s12630-021-02173-1 |
|  | Pfortmueller, C., et al. | 2017 | Acetate-buffered crystalloid infusate versus infusion of 0.9% saline and hemodynamic stability in patients undergoing renal transplantation : prospective, randomized, controlled trial | Wiener klinische Wochenschrift | 10.1007/s00508-017-1180-4 |
|  | Pfortmueller, C. A., et al. | 2015 | Balanced crystalloid use is associated with haemodynamic stability and less need for vasopressors in patients receiving renal transplantation compared to 0.9% saline | Intensive care medicine experimental | 10.1186/2197-425X-3-S1-A18 |
|  | Potura, E., et al. | 2015 | An acetate-buffered balanced crystalloid versus 0.9% saline in patients with end-stage renal disease undergoing cadaveric renal transplantation: a prospective randomized controlled trial | Anesth Analg | 10.1213/ane.0000000000000419 |
|  | Reddy, S., et al. | 2016 | Crystalloid fluid therapy | Critical Care | 10.1186/s13054-016-1217-5 |
|  | Salmela, K., et al. | 1990 | The effect of intravesically applied antibiotic solution in the prophylaxis of infectious complications of renal transplantation | Transpl Int | 10.1007/bf00333195 |
|  | Samal, S., et al. | 2019 | Balanced crystalloids in the acutely ill patient | National Medical Journal of India | 10.4103/0970-258x.291297 |
|  | Shah, R. B., et al. | 2014 | Effect of intraoperative human albumin on early graft function in renal transplantation | Saudi J Kidney Dis Transpl | 10.4103/1319-2442.144246 |
|  | Verma, B., et al. | 2016 | A multicentre, randomised controlled pilot study of fluid resuscitation with saline or Plasma-Lyte 148 in critically ill patients | Critical Care and Resuscitation | NA |
|  | Whitta, R. K., et al. | 2001 | Intraoperative mannitol does not prevent renal failure in orthotopic liver transplantation | Critical care and resuscitation | NA |
|  | Yang, L., et al. | 2024 | Dexmedetomidine use during orthotopic liver transplantation surgery on early allograft dysfunction: a randomized controlled trial | International journal of surgery (London, England) | 10.1097/JS9.0000000000001669 |
| Reduplicate cohort of Patients(n=3) | Collins, M. G., et al. | 2020 | Study Protocol for Better Evidence for Selecting Transplant Fluids (BEST-Fluids): a pragmatic, registry-based, multi-center, double-blind, randomized controlled trial evaluating the effect of intravenous fluid therapy with Plasma-Lyte 148 versus 0.9% saline on delayed graft function in deceased donor kidney transplantation | Trials | 10.1186/s13063-020-04359-2 |
|  | Collins, M. G., et al. | 2022 | Baseline Characteristics and Representativeness of Participants in the BEST-Fluids Trial: a Randomized Trial of Balanced Crystalloid Solution Versus Saline in Deceased Donor Kidney Transplantation | Transplantation direct | 10.1097/TXD.0000000000001399 |
|  | Pascoe, E. M., et al. | 2022 | Statistical analysis plan for Better Evidence for Selecting Transplant Fluids (BEST-Fluids): a randomised controlled trial of the effect of intravenous fluid therapy with balanced crystalloid versus saline on the incidence of delayed graft function in deceased donor kidney transplantation | Trials | 10.1186/s13063-021-05989-w |
| Failed to extract data(n=2) | Hayes, W. N., et al. | 2024 | A pragmatic, open-label, randomized controlled trial of Plasma-Lyte-148 versus standard intravenous fluids in children receiving kidney transplants (PLUTO) | Kidney International | 10.1016/j.kint.2023.09.032 |
|  | Paulo Do Nascimento Junior; Murilo Henrique da Veiga, F. | 2018 | Effects of Plasma-Lyte solution and 0.9% sodium chloride on post- corpus kidney transplantation: randomized clinical trial | NA | NA |
| Non-RCT(n=3) | Adwaney, A., et al. | 2017 | Perioperative Plasma-Lyte use reduces the incidence of renal replacement therapy and hyperkalaemia following renal transplantation when compared with 0.9% saline: a retrospective cohort study | Clinical Kidney Journal | 10.1093/ckj/sfx040 |
|  | Jung, S., et al. | 2022 | Effects of the Type of Intraoperative Fluid in Living Donor Kidney Transplantation: A Single-Center Retrospective Cohort Study | Yonsei Medical Journal | 10.3349/ymj.2022.63.4.380 |
|  | Nesseler, N., et al. | 2020 | Association between perioperative normal saline and delayed graft function in deceased-donor kidney transplantation: a retrospective observational study | Can J Anaesth | 10.1007/s12630-020-01577-9 |
| NA: Not Available | | | | | |

## S6 Table: Confirmation that the study was eligible to be included in the review.

| **Author, year** | **Study type** | **Type of donor** | **Main inclusion criteria** | **Intervention** | **Control** | **Group** | **cases** | **Primary outcome (s)** |
| --- | --- | --- | --- | --- | --- | --- | --- | --- |
| Collinset al. 2023 | Pragmatic,registryembedded,multicenter,double-blind,RCT | Deceased | Adults and children of any age with kidney failure admitted to a participating hospital for a deceased donor kidney transplant were eligible | Plasma-Lyte 148 | 0.9% normal saline | PL NS | 404 403 | Delayed graft function |
| doNascimentoJunior et al.2022 | Single-blinded, RCT | Deceased | Patients aged 18–65 years, of both sexes, with ASA III and IV, and on regular hemodialysis for treating ESKD, undergoing deceased kidney tranplant | Plasma-Lyte® | 0.9% saline | PL NS | 50 51 | Delayed graft function |
| 4.L. Weinberg2017 | RCT | Deceased | Adult patients (age > 18 year) undergoing deceased donor kidney transplantation | Plasma-Lyte 148 | Normal Saline | PL NS | 24 25 | Incidence of hyperkalemia within 48 h of surgery |
| Hadimioglu et al. 2008 | Prospective, doubleblinded, RCT | Living | Patients aged 18–65 year, ASA III and IV undergoing living-related kidney transplantation | Ringer’s lactate (RL) and plasmalyte (PL) | 0.9% normal saline | PL NS | 30 30 | Acid–base and electrolytes during and at the end of surgery |
| Kim et al. 2013 | Double-blinded, RCT | Living | Patients scheduled for elective living donor transplantation, aged 21–67, with ASA III to IV | Plasmalyte | Normal saline | PL NS | 30 30 | Acid–base parameters during and at the end of surgery |
| Saini et al. 2021 | Prospective, doubleblinded, RCT | Living | Patients aged 20–60 years with ASA III and IV undergoing living-related kidney transplantation | Ringer’s lactate (RL) and plasmalyte(PL) | 0.9% normal saline (NS) | PL NS | 60 60 | pH at the end of surgery |

## S7 Table: Data extraction

| **This work was performed by Yucai Chang and Yuechen Qin** | | | | |
| --- | --- | --- | --- | --- |
| **Delayed graft func-tion（DGF）** | | | | |
|  | PL | | NS | |
|  | Events | Total | Events | Total |
| Hadimioglu et al. 2008 | 1 | 30 | 3 | 30 |
| Kim et al. 2013 | 3 | 30 | 1 | 30 |
| Saini et al. 2021 | NA | 60 | NA | 60 |
| Collinset al. | 121 | 404 | 160 | 403 |
| Junior et al. 2022 | 30 | 50 | 38 | 51 |
| Weinberg et al. 2017 | 17 | 24 | 17 | 25 |
| **POD1 serum creati-nine（mg/dL）** | | | | |
|  | PL | | NS | |
|  | Mean＋SD | Total | Mean＋SD | Total |
| Hadimioglu et al. 2008 | 3.8±2.00 | 30 | 4.4±3.1 | 30 |
| Kim et al. 2013 | 3.9±2.7 | 30 | 3.6±1.6 | 30 |
| Saini et al. 2021 | 4.3±1.64 | 60 | 4.26±1.59 | 60 |
| Collinset al. | 6.37±1.71 | 404 | 6.25±2.62 | 403 |
| Weinberg et al. 2017 | 6.15±2.43 | 24 | 6.24±3.17 | 25 |
| **POD2 serum creati-nine（mg/dL）** | | | | |
|  | PL | | NS | |
|  | Mean＋SD | Total | Mean＋SD | Total |
| Hadimioglu et al. 2008 | 2.1±1.8 | 30 | 2.2±2.7 | 30 |
| Kim et al. 2013 | 1.8±2.2 | 30 | 1.6±0.6 | 30 |
| Saini et al. 2021 | 1.85±1.15 | 60 | 1.76±0.61 | 60 |
| Collinset al. | 5.21±2.61 | 403 | 5.28±2.78 | 402 |
| Weinberg et al. 2017 | 5.13±4.05 | 24 | 6.57±4.19 | 25 |
| **POD7 serum creati-nine（mg/dL）** | | | | |
|  | PL | | NS | |
|  | Mean＋SD | Total | Mean＋SD | Total |
| Hadimioglu et al. 2008 | 1.4±0.9 | 30 | 1.5 1.0 | 30 |
| Kim et al. 2013 | 1.6±1.7 | 30 | 1.3±0.7 | 30 |
| Saini et al. 2021 | 1.26±1.14 | 60 | 1.22±0.25 | 60 |
| Collinset al. | 3.76±2.67 | 398 | 4.16±3.3 | 388 |
| Weinberg et al. 2017 | 4.29±3.85 | 24 | 4.45±3.33 | 25 |
| **POD1 urine output（L)** | | | | |
|  | PL | | NS | |
|  | Mean＋SD | Total | Mean＋SD | Total |
| Hadimioglu et al. 2008 | 7.9±2.8 | 30 | 11.4±2.2 | 30 |
| Kim et al. 2013 | 9.53±3.47 | 30 | 9.78±3.65 | 30 |
| Saini et al. 2021 | 5.41±2.5 | 60 | 7.74±3.92 | 60 |
| Weinberg et al. 2017 | 1.88±3.3 | 24 | 0.55±0.63 | 25 |
| **POD2 urine output（L)** | | | | |
|  | PL | | NS | |
|  | Mean＋SD | Total | Mean＋SD | Total |
| Hadimioglu et al. 2008 | 5.4±2.5 | 30 | 7.2±3.0 | 30 |
| Kim et al. 2013 | 5.7±3.02 | 30 | 6.55±2.56 | 30 |
| Saini et al. 2021 | 4.75±2.1 | 60 | 5.0±2.6 | 60 |
| Collinset al. | 6.0±4.9 | 398 | 4.95±4.2 | 388 |
| Weinberg et al. 2017 | 1.41±1.61 | 24 | 0.83±1.34 | 25 |
| **POD7 urine output（L)** | | | | |
|  | PL | | NS | |
|  | Mean＋SD | Total | Mean＋SD | Total |
| Hadimioglu et al. 2008 | 1.7±1.4 | 30 | 2.1±1.6 | 30 |
| Kim et al. 2013 | 4.29±1.59 | 30 | 4.36±1.63 | 30 |
| Saini et al. 2021 | 3.2±1.48 | 60 | 3.32±1.21 | 60 |
| **Blood pH** | | | | |
|  | PL | | NS | |
|  | Mean＋SD | Total | Mean＋SD | Total |
| Hadimioglu et al. 2008 | 7.44±0.06 | 30 | 7.36±0.05 | 30 |
| Kim et al. 2013 | 7.41±0.049 | 30 | 7.35±0.04 | 30 |
| Saini et al. 2021 | 7.31±0.04 | 60 | 7.29±0.06 | 60 |
| Collinset al. | 7.32±0.06 | 319 | 7.27±0.06 | 312 |
| Junior et al. 2022 | 7.306±0.071 | 42 | 7.273±0.061 | 43 |
| Weinberg et al. 2017 | 7.39±0.05 | 24 | 7.32±0.06 | 25 |
| **Bicarbonate(mM/L)** | | | | |
|  | PL | | NS | |
|  | Mean＋SD | Total | Mean＋SD | Total |
| Hadimioglu et al. 2008 | 22.61±3.58 | 30 | 18.2±2.9 | 30 |
| Kim et al. 2013 | 22.54±2.94 | 30 | 19.41±3.62 | 30 |
| Saini et al. 2021 | 22.01±1.53 | 60 | 21.08±1.69 | 60 |
| Collinset al. | 22.63±2.86 | 393 | 20.6±3.01 | 399 |
| Junior et al. 2022 | 20.7±3.3 | 42 | 19.6±3.0 | 43 |
| Weinberg et al. 2017 | 23.0±4.72 | 24 | 21.0±4.32 | 25 |
| **Base excess(mEq/L)** | | | | |
|  | PL | | NS | |
|  | Mean＋SD | Total | Mean＋SD | Total |
| Hadimioglu et al. 2008 | -0.56±1.95 | 30 | -4.29±2.12 | 30 |
| Kim et al. 2013 | -2.3±3.3 | 30 | -6.0±4.2 | 30 |
| Saini et al. 2021 | -1.98±1.52 | 60 | -2.98±1.78 | 60 |
| Junior et al. 2022 | -4.6±4.3 | 42 | -6.0±4.5 | 43 |
| **Chloride(mM/L)** | | | | |
|  | PL | | NS | |
|  | Mean＋SD | Total | Mean＋SD | Total |
| Hadimioglu et al. 2008 | 106.5±2.2 | 30 | 125.4±3.7 | 30 |
| Kim et al. 2013 | 100.05±5.02 | 30 | 105.2±4.63 | 30 |
| Saini et al. 2021 | 105.3±1.96 | 60 | 118.49±4.47 | 60 |
| Collinset al. | 96.38±4.17 | 392 | 102.46±4.68 | 395 |
| Junior et al. 2022 | 99.6±4.2 | 42 | 103.3±5.6 | 43 |
| Weinberg et al. 2017 | 96.3±3.0 | 24 | 102.3±4.8 | 25 |
| **Sodium (mEq/L)** | | | | |
|  | PL | | NS | |
|  | Mean＋SD | Total | Mean＋SD | Total |
| Kim et al. 2013 | 134.35±3.82 | 30 | 136.61±2.96 | 30 |
| Collinset al. | 135.4±3.69 | 401 | 136.39±3.55 | 401 |
| Junior et al. 2022 | 134.8±3.6 | 42 | 135.7±4.6 | 43 |
| Weinberg et al. 2017 | 138.7±2.9 | 24 | 139.0±2.8 | 25 |
| **Potassium** | | | | |
|  | PL | | NS | |
|  | Mean＋SD | Total | Mean＋SD | Total |
| Hadimioglu et al. 2008 | 3.8±0.25 | 30 | 3.9±0.4 | 30 |
| Saini et al. 2021 | 4.04±0.63 | 60 | 3.8±0.54 | 60 |
| Collinset al. | 5.0±0.76 | 401 | 5.05±0.88 | 400 |
| Junior et al. 2022 | 5.1±0.9 | 42 | 5.1±0.8 | 43 |
| Weinberg et al. 2017 | 5.0±0.9 | 24 | 5.6±1.1 | 25 |
| NA: Not Available; NS: normal saline, PL: plasma-lyte; SD: standard deviation  POD: postoperative days | | | | |

# Figures

## S1 Fig: Forest plot of subgroup according to the type of donor for DGF

**
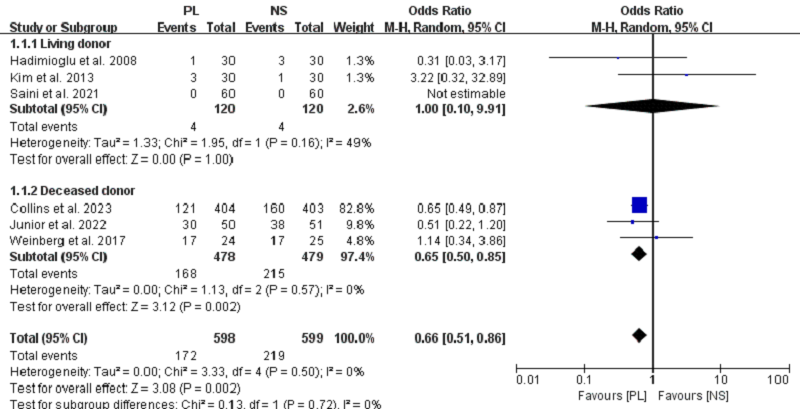
**

**OR: odd ratio, CI: confidence interval**

## S2 Fig: : Forest plot of subgroup according to the type of donor

### S2 Fig A: Forest plot of subgroup according to the type of donor for POD1 serum creatinine


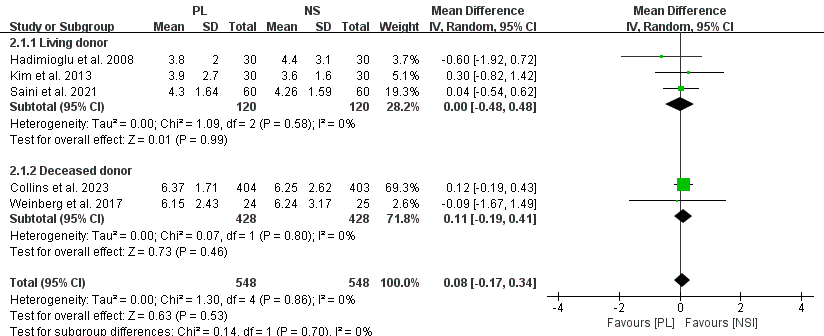


**MD: mean difference, CI: confidence interval, POD: postoperative days**

### **S2 Fig B: Forest plot of subgroup according to the type of donor for POD2 serum creatinine**
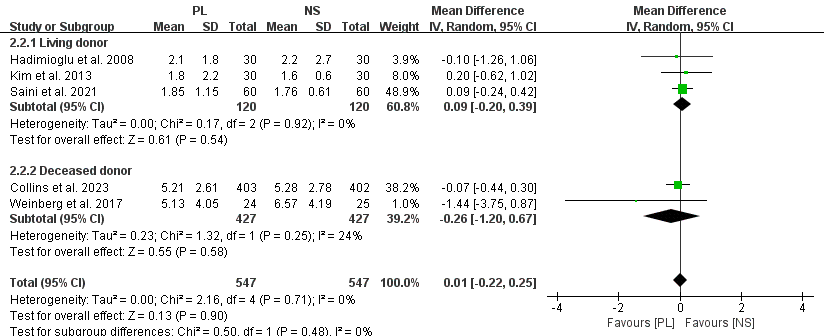


**MD: mean difference, CI: confidence interval, POD: postoperative days**

### S2 Fig C: Forest plot of subgroup according to the type of donor for POD7 serum creatinine


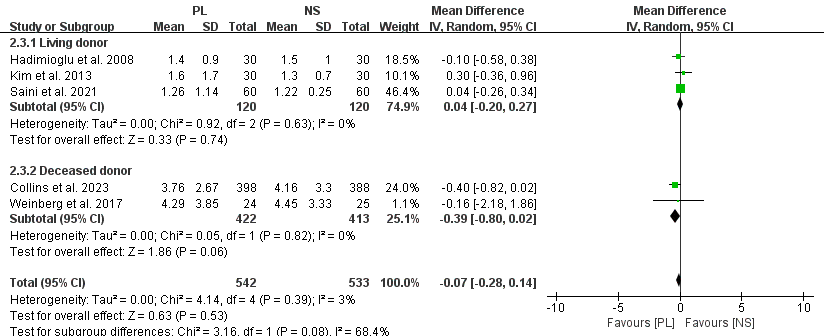


**MD: mean difference, CI: confidence interval, POD: postoperative days**

## **S3 Fig: Forest plot of subgroup according to the type of donor**

### **S3 Fig A: Forest plot of subgroup according to the type of donor for POD1 urine output**
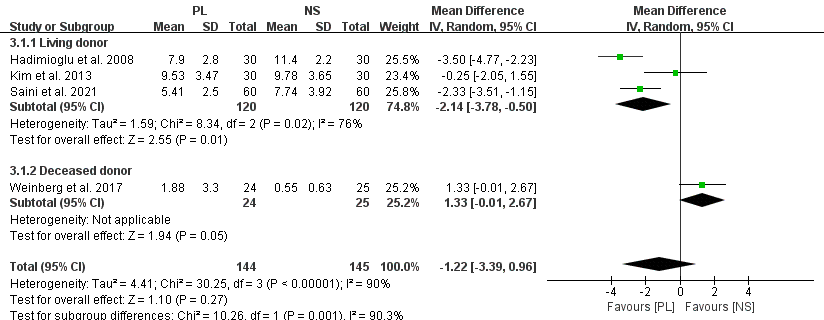


**MD: mean difference, CI: confidence interval, POD: postoperative days**

### S3 Fig B: Forest plot of subgroup according to the type of donor for POD2 urine output
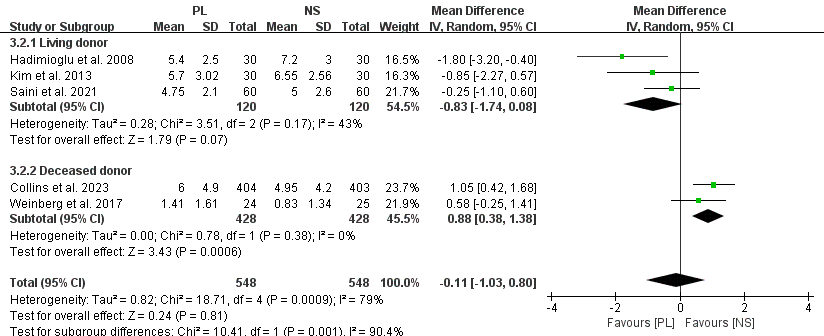


**MD: mean difference, CI: confidence interval, POD: postoperative days**

### S3 Fig C: Forest plot of subgroup according to the type of donor for POD7 urine output
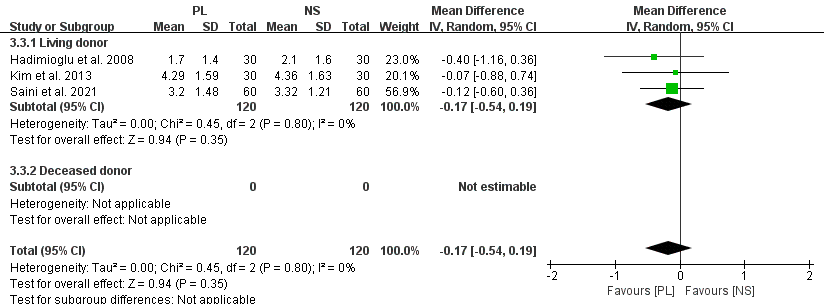


**MD: mean difference, CI: confidence interval, POD: postoperative days**

## ****S4 Fig: Forest plot of subgroup according to the type of donor for Blood pH****


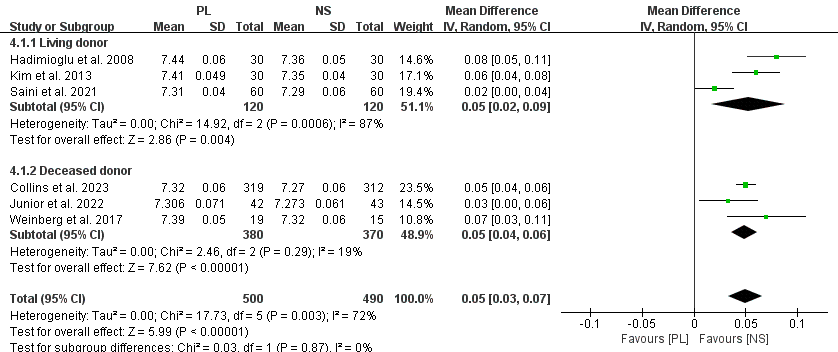


**MD: mean difference, CI: confidence interval**

## ****S5 Fig: Forest plot of subgroup according to the type of donor for Bicarbonate levels****


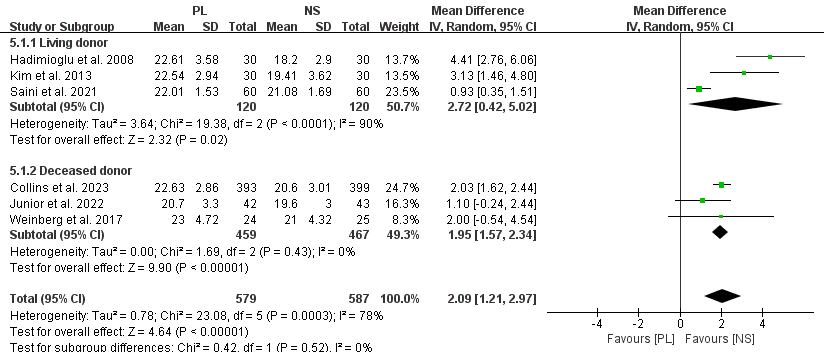


**MD: mean difference, CI: confidence interval**

## ****S6 Fig: Forest plot of subgroup according to the type of donor for Base excess****


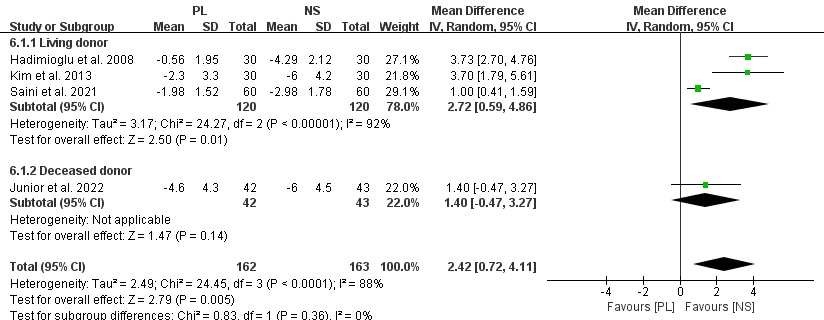


**MD: mean difference, CI: confidence interval**

## S7 Fig: Forest plot of subgroup according to the type of donor for Serum chloride
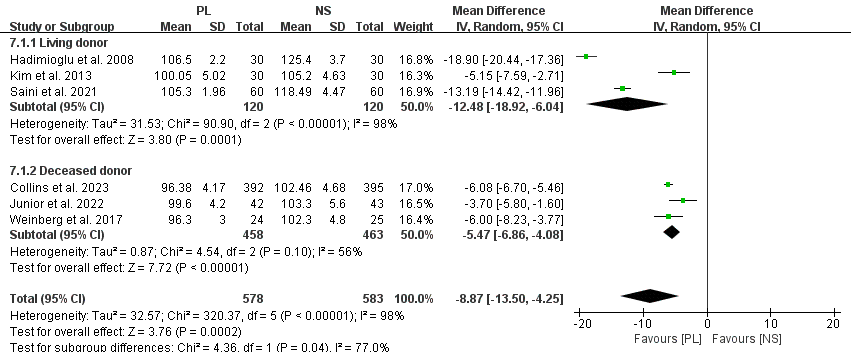


**MD: mean difference, CI: confidence interval**

## S8 Fig: Forest plot of subgroup according to the type of donor for Serum sodium
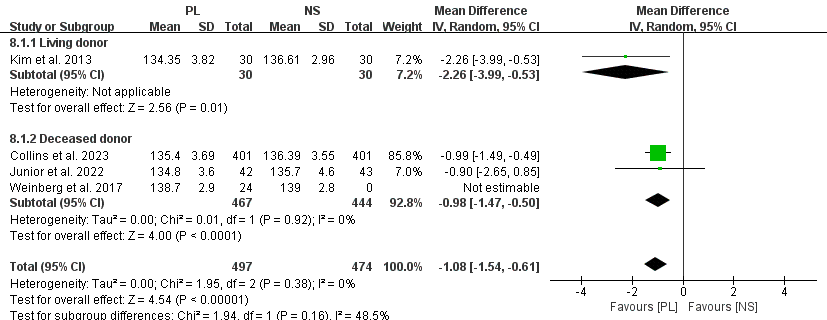


**MD: mean difference, CI: confidence interval**

## S9 Fig: Forest plot of subgroup according to the type of donor for Serum potassium
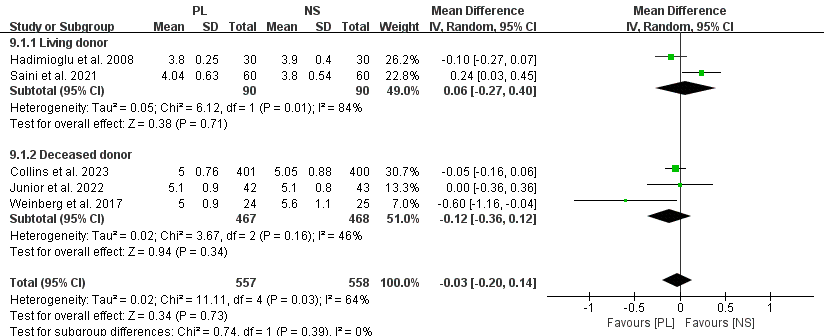


**MD: mean difference, CI: confidence interval**
